# Synergistic magneto-enzymatic propulsion enables multimodal nanomotor swarms

**DOI:** 10.1038/s41467-026-76053-x

**Published:** 2026-08-11

**Authors:** Anna C. Bakenecker, Xavier Arqué, Xander Peetroons, Paul E. D. Soto Rodriguez, Sandra Prévéral, Damien Faivre, Samuel Sánchez

**Affiliations:** 1 Institute for Bioengineering of Catalonia (IBEC), Barcelona Institute of Science and Technology (BIST), Barcelona, Spain; 2 Faculty of Pharmacy and Food Sciences, University of Barcelona, Barcelona, Spain; 3 Aix-Marseille University, CNRS, CEA, BIAM, Saint-Paul-lez-Durance, France; 4 Instituto de estudios avanzados IUDEA, Departamento de Física, Universidad de La Laguna C/Astrofísico Francisco Sánchez, Tenerife, Spain; 5 Department of Physics, Faculty of Science and Technology, University of Latvia, Riga, Latvia; 6 Catalan Institute for Research and Advanced Studies (ICREA), Barcelona, Spain; 7 Present Address: Medical Engineering, Department of Electrical Engineering and Information Technology, Technical University of Darmstadt, Darmstadt, Germany; 8 Present Address: Gulliver UMR CNRS 7083, ESPCI ParisTech, PSL Research University, Paris, France

**Keywords:** Nanoparticles, Magnetic properties and materials

## Abstract

Enzymatic nanomotors self-propel by converting free molecular energy from chemical reactions. While initially used mainly as individual particles, these systems are increasingly implemented as swarms, where collective effects enable adaptable navigation in complex environments, broader coverage and enhanced local agent concentration. However, most studies have focused on planar swarming, and collective systems that combine magnetic navigation with enzyme-powered motion remain largely underexplored. Here, as a mechanistic proof-of-principle, we introduce urease-powered nanomotors based on bioproduced magnetosomes that operate in two sequential collective modes: (i) magnetic localization of the ensemble and (ii) catalysis-triggered, interface-dependent expansion of that localized cloud. We investigate this catalytic collective phenomenon under varying conditions, including particle and substrate concentrations, media viscosity, and confinement, and devote particular attention to distinguishing urea-driven buoyant accumulation from catalysis-dependent interfacial spreading using combined top/side-view imaging and inactive particle controls. Finally, we examine the swarm under a magnetic field gradient to concentrate it in a target area and then trigger catalytic expansion. In confined environments, where the free-interface expansion is suppressed, magnetic localization and local enrichment remain operative. Rather than targeting a specific immediate application, this work establishes a two step collective strategy, clarifies the mechanism underlying a distinct catalytic swarm mode, and defines the physical conditions that may support future environmental and biomedical implementations of multimodal nanomotor swarms.

## Abstract (web summary)

Enzyme-powered nanomotors are primarily studied as individual particles with limited understanding of their collective behaviors, especially in three-dimensional and interface-dependent environments. This study reports a mechanistic proof-of-principle for a two-step multimodal nanomotor swarm that combines magnetic localization with catalysis-triggered interfacial expansion, elucidating the physical conditions governing their collective dynamics.

## Introduction

Inspired by the active motion of biological molecular motors, nanoscale motile systems, which are commonly referred to as nanomachines or nanomotors, have garnered increasing interest over the past few decades^1^. Among the various propulsion engines, enzymatic micro- and nanomotors stand out for their ability to self-propel by converting free molecular energy from in situ chemical reactions^2–4^, while offering notable advantages in terms of biocompatibility, substrate versatility, and bioavailability^5–8^. Catalytic motors can modulate the physicochemical properties of their surroundings, such as pH or viscosity^9,10^, enabling them to overcome obstacles like biological barriers and penetrate target areas more effectively^11–13^. These unique capabilities make enzyme-powered motors particularly appealing for biomedical applications^14,15^, where therapeutic agents must reach localized and hard-to-access sites^16^. Beyond healthcare, their catalytic and micromixing properties have applications in compound sensing, water decontamination, and antimicrobial treatments^17,18^.

Despite two decades of research, most studies have focused on the motion dynamics of individual enzyme-powered motors^6,19,20^. However, these devices hold great promise when deployed collectively as swarms^21,22^. Hence, expanding their operational range, amplifying their impact on target areas, enabling therapeutically relevant dosages, and displaying novel behaviors that emerge only in large populations^23–25^. In the context of active nanomotors, we use the term swarming in the broader active matter sense to denote a global collective behavior without centralized control, in which the dynamics of the ensemble emerge through coupling with the surrounding physicochemical environment rather than necessarily through direct communication between individual particles. Collective swarms of nanomotors have demonstrated complex behaviors, such as clustering, beating dynamics^26,27^, and predator-prey interactions^28,29^, as well as reconfigurable, on-demand motion patterns^30–33^. However, most of these promising capabilities are reported from a 2D analysis, offering limited insight into 3D dynamics. Only recent efforts have started exploring swarming behavior under different spatial configurations to provide a more comprehensive understanding of swarm mechanisms^34,35^.

Notably, 3D collective motion has been proven in vivo through positron emission tomography (PET) detection of urease nanomotors^36^, an attractive system given its enhanced penetration reported in vitro^10,13^ and in vivo^37^. Such urease-powered nanomotors have shown particular promise^38^ in scenarios where urea is abundant, such as in the urinary tract^39,40^, or where a rise in pH is beneficial^9^, like in certain antimicrobial treatments^41^. However, the collective mode addressed in the present work is specifically an interface-dependent one and is therefore distinct from previously reported urease swarm behavior in fully liquid-filled in vivo environments, such as the bladder. Rather than claiming direct, immediate in vivo translation of this specific interfacial mode, we use these prior studies to motivate the broader relevance of understanding how urease-powered swarms can display distinct collective behavior under different physicochemical conditions.

Nevertheless, enzyme-powered active motion lacks directional command. Although there are biochemical strategies to target specific areas^42,43^, physical guidance, such as magnetic navigation, could significantly enhance their effectiveness by enabling precise targeting while reducing the required dosage. In this regard, reconfigurable and adaptive collective motion controlled on-demand has been achieved recently through magnetic^15,33,44–48^ and light^49,50^ stimulation of micro- and nanomotors, but primarily reporting navigation in 2D studies from a top view.

While magnetic navigation of micro- and nanomotors has been widely studied over the past years^51,52^, it has been combined a number of times with catalytic capabilities to synergistically boost their features as single motors^8,53–61^, often using magnetic fields as guidance for enzymatic propulsion and, to our knowledge, never as magneto-catalytic swarms. Despite being a consolidated and spread technique, magnetic navigation is generally based on magnetically responsive materials that present problems of monodispersity, toxicity, and biocompatibility^62–64^. Under this framework, the biomineralization of magnetosomes inside magnetotactic bacteria^65^ offers a promising alternative source of magneto-responsive particles to be used as a natural and biocompatible motor chassis with uniform nanometric size and shape^66,67^.

Herein, we present a urease-powered nanomotor based on bioproduced magnetosomes as a mechanistic proof-of-principle of a two-step collective strategy. In the first mode, the ensemble is magnetically localized at a selected region. In a second, interface-dependent mode, ureolysis triggers a Marangoni-driven expansion that redistributes the localized cloud. Throughout this work, we use the term multimodal to refer to these distinct collective operating modes of the swarm, rather than to multiple independent propulsion engines at the single-particle level. We study how particle loading, urea concentration, viscosity, and confinement govern the emergence or quenching of this catalytic expansion.

We have considered often-dismissed aspects of collective active matter, such as the observation of the behavior both from a top and a side view to consider lateral motion and therefore indirectly assess 3D motion, as well as the investigation of motion phenomena of inactive nanoparticles in urea (substrate) to separate buoyant accumulation from true catalytic interfacial spreading. In this sense, the collective behavior studied here is not based on direct particle-particle communication, but on ensemble-level dynamics that emerge through particle cloud-environment coupling under catalytic, interfacial, buoyant, and magnetic influences.

**Fig. 1:**
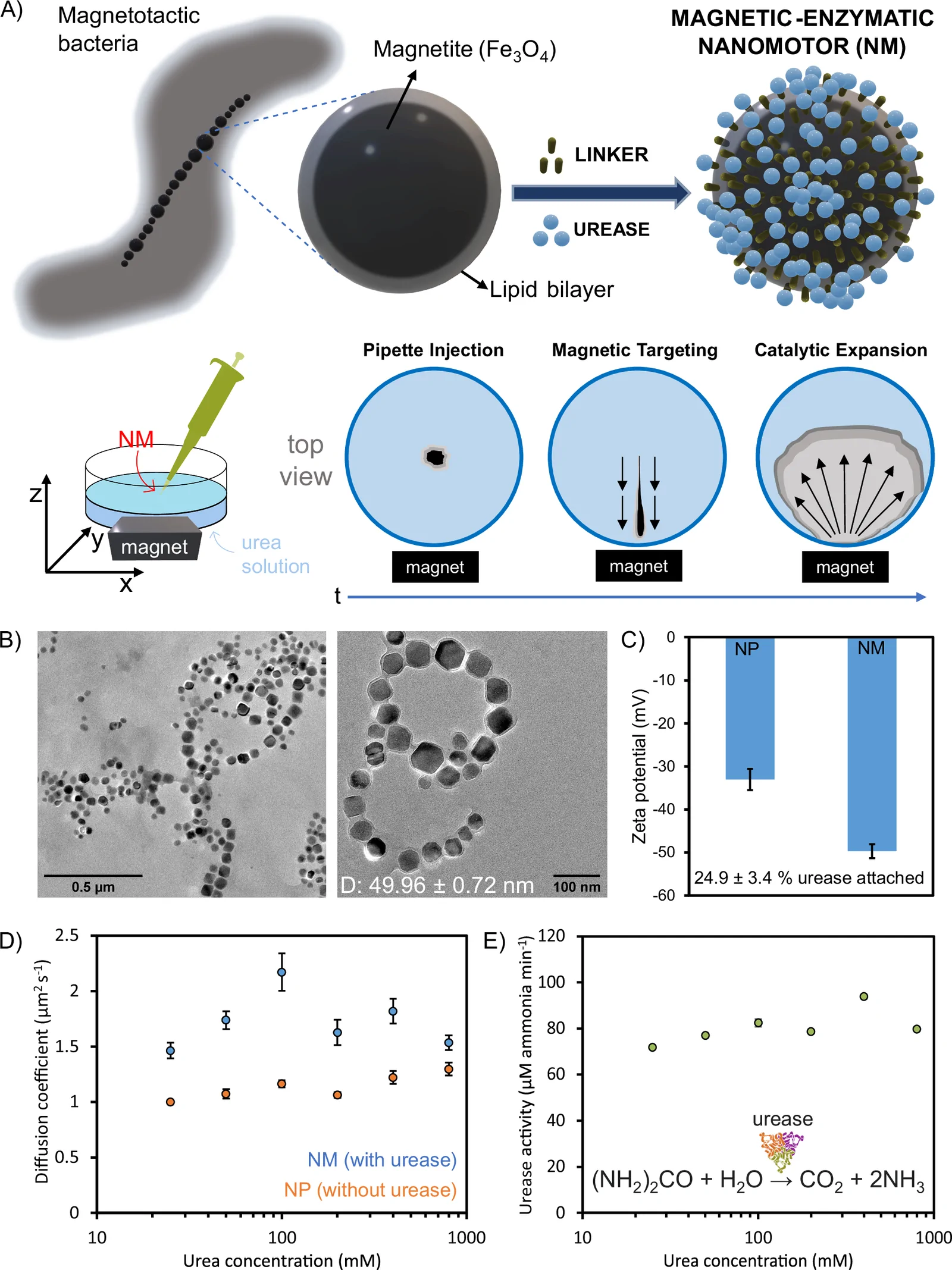
Nanomotor system schematic, fabrication, and characterization of urease-magnetosome nanomotors. **A** Schematic of magnetotactic bacteria from which magnetosomes are extracted to fabricate magnetosome-based nanomotors by attaching urease. Proposed nanomotor swarm operating principle: the particle cloud is magnetically localized at the target region before undergoing catalysis-triggered interfacial expansion. **B** TEM micrographs of magnetosome crystals (diameter calculated from *N* = 252). **C** Measurement of zeta potential of non-functionalized (NP) and urease-functionalized nanomotors (NM). Inset: % of urease attached from the initial 3 mg ⋅ mL^−1^, measured through total protein quantification. **D** Diffusion coefficient extracted from dynamic light scattering (DLS) for magnetosomes with (NM) and without (NP) urease functionalization and for different concentrations of urea. **E** Enzymatic activity of urease nanomotors for different concentrations of urea. All results are shown as the mean  ± standard error of the mean.

The swarming dynamics are computationally analyzed by different parameters, such as the expansion and vertical and horizontal movement of the nanomotor clouds, using a self-written code. Finally, we analyze the collective behavior in a magnetic field to steer the swarm through open and confined settings towards a target area and then trigger the catalytic expansion to cover it, always controlling for the effect of catalytic activity and fuel presence in the media. Beyond the identification of a distinct catalytic swarm mechanism, this hybrid strategy also enables a non-trivial combination of directionality and target-area coverage: the swarm can first be guided to a selected location and then expand over that target once it reaches the interface. As summarized schematically in Fig. 1A, the purpose of the present work is to test under which physicochemical conditions this proposed collective sequence emerges. This work identifies the governing balances and threshold conditions for a controllable magneto-enzymatic collective state and examines how the combined directional and expansive swarm behavior is enabled in open environments and hindered under increasing confinement. Such controllable collective behavior is of broad interest as a first step toward future environmental and biomedical nanomotor applications.

## Results and discussion

### Characterization of urease-powered magnetosome nanomotors

Magnetotactic bacteria naturally align with magnetic fields due to the biomineralization of iron oxide nanoparticles in a lipid bilayer within their cytoplasm^65^. Firstly, these magnetotactic organelles, known as magnetosomes, are extracted from *Magnetospirillum magneticum* AMB-1 (Fig. 1A; see Methods). After isolation, we characterized the magnetosomes with transmission electron microscopy (TEM) (Fig. 1B; see Methods), obtaining an average diameter of 49.96 ± 0.72 nm (*N* = 252).

The urease-magnetosome nanomotors (NM) are fabricated by functionalizing the lipid bilayer of the iron oxide core (Fe_3_O_4_) of the magnetosomes with urease (Fig. 1A; see Methods). Urease attaches to the magnetosome surface via non-covalent interactions, such as electrostatic forces, van der Waals interactions, and hydrogen bonding. Successful enzyme attachment is confirmed by a zeta potential shift from  −33.03 ± 0.24 mV for bare magnetosomes to  −49.7 ± 0.7 mV after urease incorporation (Fig. 1C; see Methods). Protein quantification reveals that 24.9 ± 3.4% of the initial 3 mg ⋅ mL^−1^ urease solution is adsorbed onto the magnetosome nanoparticles (see Methods). The enzymatic functionality of the NM is evaluated by measuring its catalytic activity with varying urea concentrations (25, 50, 100, 200, 400, and 800 mM urea). Urease catalyzes urea into ammonia and carbon dioxide via the following reaction:

1 $$({\mathrm{NH}}_{2})_2{\mathrm{CO}}+{\mathrm{H}}_2{\mathrm{O}} \mathop{\longrightarrow}\limits^{{\mathrm{urease}}}{\mathrm{CO}}_2+2{\mathrm{NH}}_3.$$

By measuring the generated ammonia, we observe increased enzymatic activity with higher urea concentrations, which exhibits a plateau characteristic of Michaelis-Menten kinetics (Fig. 1E; see Methods).

Next, we characterize the active motion capabilities of the NM by dynamic light scattering (DLS) (Fig. 1D). The diffusion coefficient is assessed for varying urea concentrations (25, 50, 100, 200, 400, and 800 mM urea) with and without urease attached (see Methods). In the absence of urease, the particles exhibit base Brownian motion with a diffusion coefficient of 1.1 μm^2^ ⋅ s^−1^, increasing slightly to 1.3 μm^2^ ⋅ s^−1^ at higher urea concentrations, likely due to enhanced fluid dynamics and increasing buoyancy. However, when urease is present, a more pronounced enhancement of diffusion is observed from substrate concentrations of 25 mM urea (1.5 μm^2^ ⋅ s^−1^) to 100 mM (2.2 μm^2^ ⋅ s^−1^). This enhancement diminishes at higher urea concentrations, suggesting the substrate increase induces a higher viscosity since enzymatic activity is independently stable. These observations support previous findings on enzymatic activity driving propulsion and resulting motion depending on media viscosity^6,38^.

Although the asymmetric urease coating can in principle support active motion, the goal of the present work is not to maximize persistent single-particle propulsion, but rather to understand the ensemble-level collective behavior that emerges in this system. At the nanoscale, the effective diffusion of an active particle reflects not only catalytic propulsion, but also its passive Brownian motion and, importantly, its orientational persistence. In very small particles, the passive diffusivity is relatively large, but rotational randomization is also much faster, so directional persistence is rapidly lost. As a result, even when catalytic asymmetry is present, a 50 nm particle can still display only a modest enhancement over Brownian diffusion, consistent with previous reports on urease-powered nanomotors showing comparable diffusion enhancement^42,68,69^. Importantly, the focus of this work is not strong single-particle motility, but rather the emergence of functionally useful collective dynamics from limited individual nanoscale motion.

### Optimization of swarming conditions for urease-magnetosome nanomotors

**Fig. 2:**
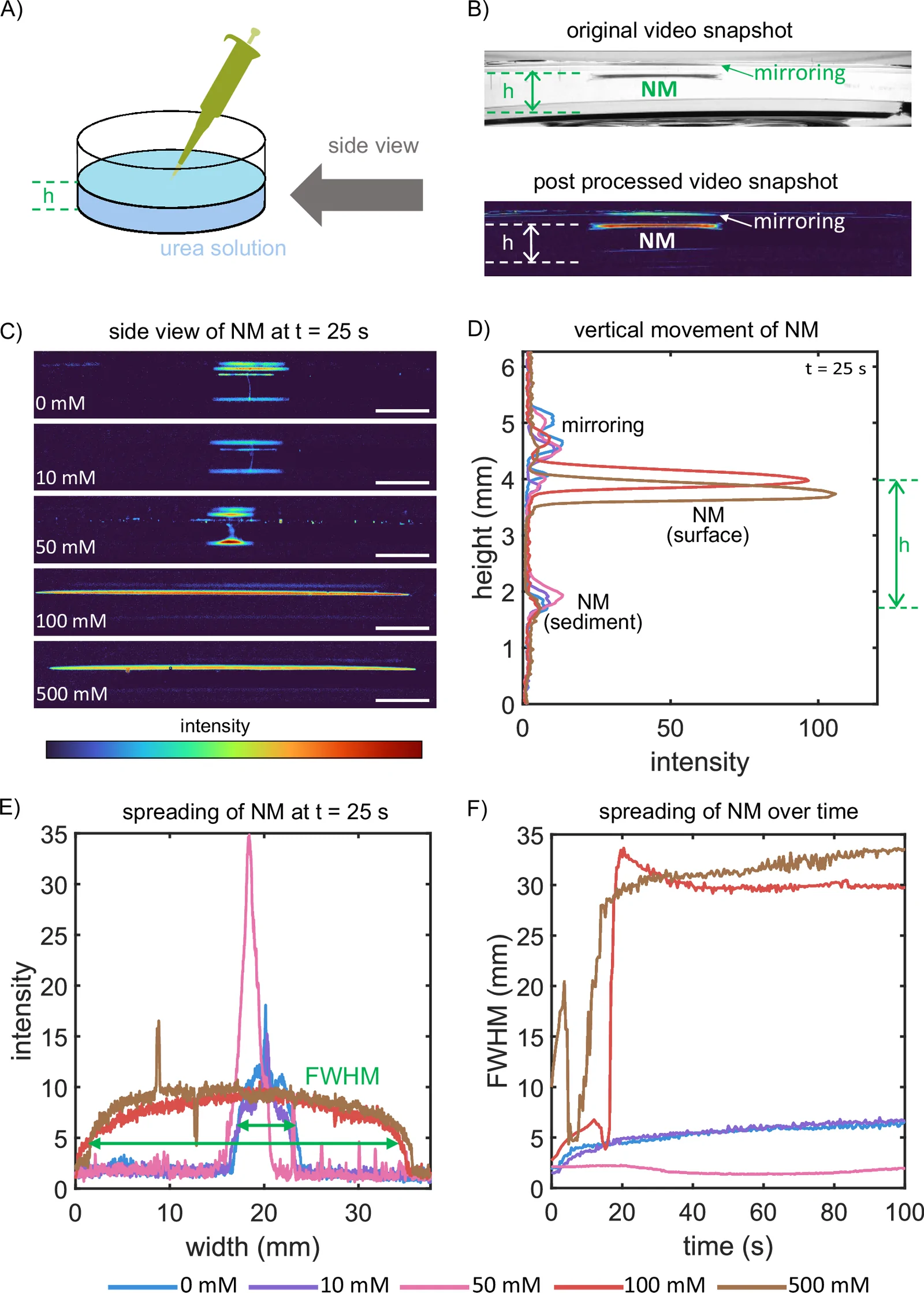
Characterization of collective motion behavior of urease-magnetosome nanomotors (NM) for different concentrations of urea. **A** Schematic of the experimental setup based on a Petri dish filled with urea solution where the NM are pipetted and recorded from the side view. **B** Original side-view video snapshots recorded in gray scale and then post-processed and displayed with colormap, h indicates the hight of the solution, a mirroring effect occurs on the surface. **C** NM swarms at different urea concentrations (0, 10, 50, 100, 500 mM urea). Representative snapshots of the side-view videos at *t* = 25 s from addition of swarms for different urea concentrations (Video 1). Scale bar 5 mm. **D** Intensity projections of the vertical movement of the NM at *t* = 25 s for different urea concentrations. **E** Intensity projections of the lateral movements of NM at *t* = 25 s for different urea concentrations. **F** Quantification of the dissipation through FWHM over time of the vertical intensity projections for different urea concentrations.

Following the characterization of individual NM motion, we assess the collective behavior. A droplet of NM suspension in water is introduced into a Petri dish containing urea solution, and the behavior is recorded from both top and side views to better understand the collective dynamics in X-, Y-, and Z-directions (Fig. 2A). Then, the videos are post-processed and color-coded, with horizontal and vertical intensity projections calculated using a self-written MATLAB program to analyze swarm expansion. Full width at half maximum (FWHM) values are calculated to quantify the swarm diameter as the particles expand isotropically (see Methods).

First, we evaluate the effect of varying urea concentrations on swarm behavior (Fig. 2, Fig. S1, and Video 1). We study the phenomenon through a side view, and we calculate the intensity projections of the video frames by first subtracting the static background from the recorded videos (Fig. 2B). The side-view snapshots in Fig. 2C reveal that active NM show significant expansion only at urea concentrations of 100 mM or higher. At such conditions, NM moves upward to the air-water interface, while sedimentation occurs at lower urea concentrations. Horizontal intensity projections (Fig. 2D) further confirm that NM only displays upward and expansive motion at urea concentrations equal or higher than 100 mM. Remarkably, similar upward motion is observed for non-functionalized magnetosome nanoparticles (NP) at very high urea concentrations (500 mM) (Fig. S1), suggesting a buoyancy effect that we explore further in Section 2.3. At this concentration, the NP cloud reaches the interface already broadly distributed, but only the active NM exhibits an evolving expansive behavior at the interface. This behavior can also be appreciated in Video 1, where the 500 mM NP condition shows immediate broad interfacial accumulation without the later front-like expansion observed for active NM in the same video.

The side-view analysis reveals an isotropic expansion upon reaching the air-water interface (Fig. 2E). Horizontal intensity projections quantify the swarm expansion in X- and Y-directions, showing a clear dependence on higher urea concentration. At 100 mM urea, NM displays significant expansion after 20 s, while at 500 mM, the expansion begins immediately upon NM addition (Fig. 2F), making the system difficult to control. In all cases a part of the sample sediments at the bottom, but less for higher urea concentrations. The 50 mM condition appears to lie in an intermediate buoyancy regime, where the nanomotor population does not reorganize into a fully sedimented or a fully uplifted cloud. The data suggest the coexistence of two subpopulations: one that still sediments and another that experiences partial uplift. This leads to a more limited overall spreading, while maintaining a relatively thin and concentrated signal distribution for longer times, which is consistent with the pronounced intensity peak observed in Fig. 2C–E. This transitional behavior can also be appreciated in Video 1, where the 50 mM condition shows less coherent upward reorganization than the higher urea concentrations. For subsequent experiments, we select 100 mM urea as the optimal concentration based on the aforementioned DLS and swarming results, hence providing a 20 s latency window to exploit later for magnetic steering before the catalytic expansion occurs.

Next, we examine the effect of NM concentration on swarm dynamics from a top view (Fig. S2 and Video 2). At 100 mM urea, the optimal NM concentration produces an abrupt and extensive swarm expansion observed in prior experiments (Fig. S2B). FWHM analysis shows a slow spread for the initial 20 s since seeding, followed by a rapid expansion lasting 2 s (Fig. S2C). During this phase, the swarm diameter increases from 2 to 20 mm, followed by a plateau of 21 mm. Higher NM concentrations lead to sedimentation of the nanomotor cloud, hindering the collective behavior probably due to strong dipole-dipole interactions between magnetosomes, and maintaining an area explored at a diameter of 3 mm. In contrast, lower particle concentrations result in dispersed and less expansive motion, likely due to a weakening of the catalytically induced expansion at the air-water interface, suggesting that NM only move as individuals at low concentrations. Hence, the fewer nanomotors added in solution, the smaller the area explored by their expansion, with no condition reaching a FWHM larger than 9 mm.

Finally, we assess the impact of viscosity on swarming dynamics (Fig. S3 and Video 3). Using optimal NM and fuel concentrations, we add glycerol to the medium to increase viscosity (from 0.91 to 1.18 mPa ⋅ s) and record the side-view behavior (Fig. S3A). Higher viscosity quenches the catalytic expansion, as observed in the snapshots at *t* = 25 s (Fig. S3B and Video 3) and their respective vertical intensity projections (Fig. S3C). This behavior is consistent with a Marangoni-driven mechanism, because the interfacial velocity decreases with increasing viscosity and glycerol can additionally increase the effective interfacial viscosity/elasticity, thereby weakening the surface-tension gradients that sustain the front. FWHM analysis reveals slower expansion rates and smaller maximum diameters when increasing glycerol concentrations: 33 mm at lowest viscosity of 0.91 mPa ⋅ s and reaching 14 mm at the highest viscosity of 1.18 mPa ⋅ s. The spreading of inactive NM and NP (without fuel) increases slightly with viscosity, given the increase in buoyancy (Fig. S4), hence showing broader distributions instead of sedimentation. This behavior is different from the sudden catalytic expansion of active NM in urea, since the broadening is monotonously increasing and the maximum diameter reaches only 14 mm. By contrast, the control conditions in Fig. S4 show only modest broadening with increasing viscosity, consistent with reduced sedimentation and longer interfacial residence, rather than a sharp catalytic front. These findings establish the optimal conditions of fuel concentration, particle concentration, and viscosity for urease-powered NM swarming.

### Expansive swarming behavior of urease-magnetosome nanomotors

**Fig. 3:**
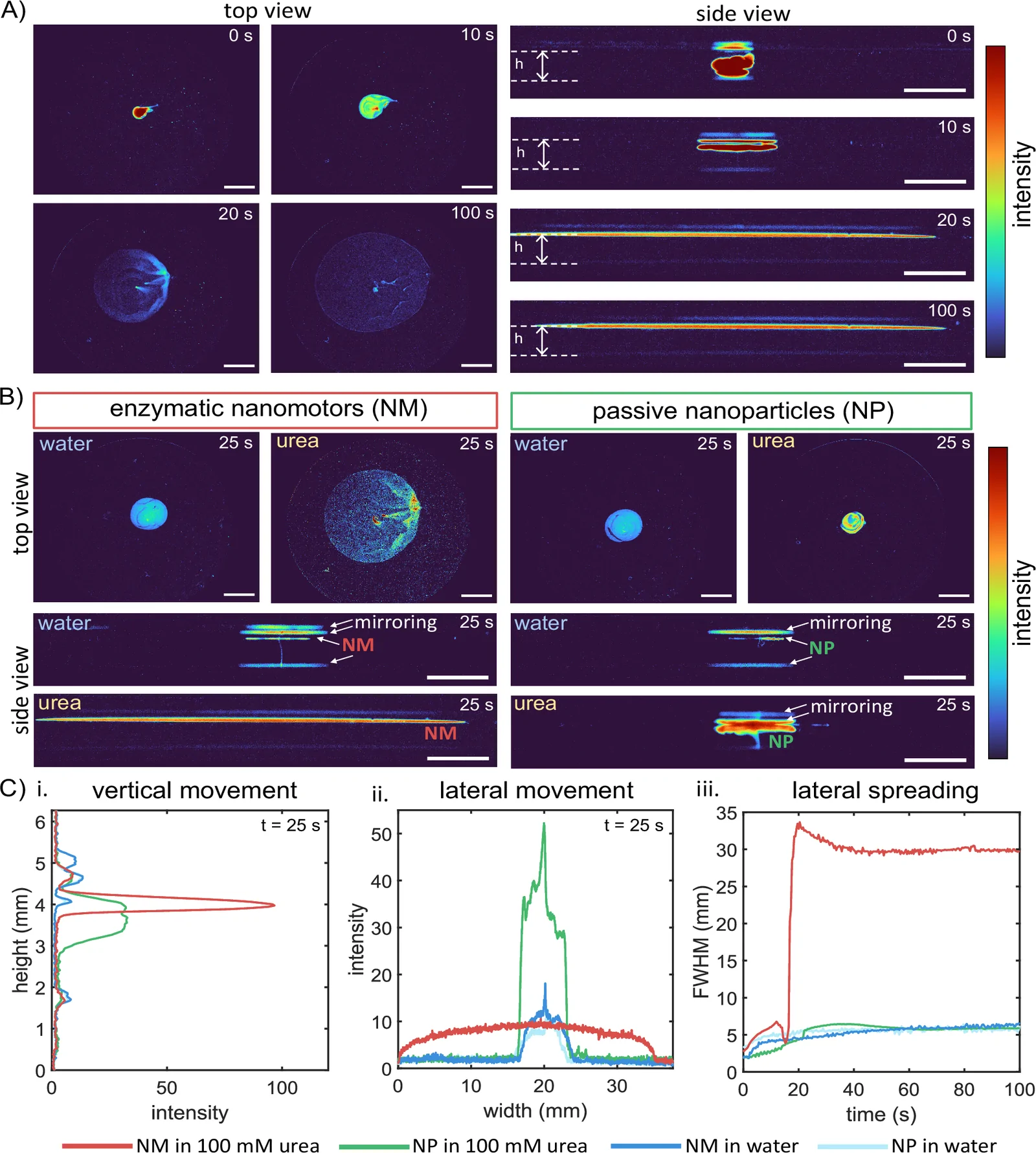
Collective behavior of urease-magnetosome nanomotors. **A** Representative snapshots of the top view and side view videos at indicated time points (0, 10, 20, 100 s) of the NM in 100 mM urea (Video 4) **B** Representative snapshots of top view and side view of NM and NP in water and 100 mM urea at *t* = 25 s within a controlled height (h). **C** Computational analysis: Intensity projections of the side view video frames at 25 s indicating i. the vertical and ii. the lateral movement of the NM and iii. the derived FWHM over time of the lateral movement. All scale bars 5 mm, false colors display the intensity.

After optimizing the swarm conditions, we study in detail the evolution of the collective expansive behavior both from a top and a side view (Fig. 3). These expansive dynamics at the air-water interface are compared to the controls of NM in water and inactive NP (i.e., non-functionalized) in urea and water. All the videos are post-processed (i.e., background subtraction and colormap representation), and the intensity projections and FWHM are calculated.

From the top-view snapshots at different time points (0, 10, 20, and 100 s) (Fig. 3A left, Video 4), we conclude that the NM in urea show an overall broader dissipation over time compared to NM in water (0 mM), and NP in urea (100 mM) or water (0 mM) (Fig. 3B). Initially, from this information, it is clear that urease decomposition of urea into ammonia and carbon dioxide plays a key role in the expansive behavior of the NM. From the side-view video snapshots at 25 s after seeding (Fig. 3B bottom and Video 4), we conclude that the NM and NP in urea move towards the surface of the media due to different densities and higher buoyancy, while they sediment in water (0 mM urea). Once the NM in urea reaches the surface of the medium, they immediately spread, while the NP in urea stays below the surface. From these combined observations, it becomes clear that two distinct processes contribute to the collective behavior. First, urea establishes vertical concentration gradients that promote upward accumulation of both active NM and passive NP at the air-water interface, whereas in water both systems sediment. Second, only the active NM undergoes an abrupt interfacial expansion once this accumulated cloud reaches the surface. We therefore attribute the rapid spreading not to buoyancy alone, but to a catalysis-dependent interfacial mechanism. Specifically, ureolysis generates ${{{\rm{NH}}}_{4}}^{+}$ OH^−^, and bicarbonate/carbonate species, and locally raises the pH. We propose that this local change in pH and ionic composition alters the protonation state and interfacial activity of endogenous amphiphilic components of the magnetosome membrane, including phospholipids and acidic magnetosome-associated proteins. The resulting surface-tension gradients drive outward Marangoni flow, producing the lateral expansion along the interface. In all side-view experiment,s there is a small portion of sample sedimentation, as well as a mirroring effect that appears as signal above the air-water interface. Recent work shows that free urease can generate interfacial Marangoni flow purely via its amphiphilicity, without requiring catalysis^70^. In contrast, our magnetosome system requires the ureolysis to sustain the strong interfacial gradient: inactive NP in urea undergoes uplift, but no abrupt front. This is consistent with silica-based urease nanomotors, which lack membrane amphiphiles and display urea-induced buoyant rise but no interfacial expansion^34^. This distinction is already visible in the control condition shown in Fig. S1, where NP at 500 mM urea accumulates broadly at the interface without developing the later abrupt spreading observed for active NM.

This interpretation is also consistent with previous urease-powered micro/nanomotor studies in which catalytic activity gives rise to local pH changes or pH-mediated functional effects. For example, Patiño et al. used pH-responsive DNA nanoswitches to sense and respond to the local alkalinization generated by ureolytic activity^71^, while Arqué et al. showed that the ionic species produced during urea hydrolysis play a central role in propulsion and environmental modulation^9^. In a related biomedical setting, Choi et al. showed that urease micromotors can locally increase pH and thereby induce gel-sol transition of the mucus layer to facilitate penetration^72^. Most importantly in the present context, Chen et al. demonstrated that urease swarms display collective behavior associated with the products of ureolysis in a buoyancy-driven mechanism^34^. Although the interface-dependent Marangoni mechanism reported here is distinct, these studies provide a clear precedent for pH modulation as a functionally relevant mechanism in urease-powered systems, including at the collective/swarm level.

To further support this interpretation under the experimental conditions used here, we monitored the pH evolution of the relevant control and catalytic systems (Fig. S5). The addition of NM to 100 mM urea produced a clear pH increase above 8.5, whereas 100 mM urea alone and magnetosomes in water showed only minor pH variations of 6.6-6.8. Because this measurement reports the bulk pH of the solution, the rise observed over 15–20 min likely underestimates the faster and higher local alkalinization expected in the immediate vicinity of the NM and at the air-water interface during the early stages of swarm expansion. Moreover, after swarm operation, particle recovery, and washing, the recovered NM still induced a clear pH increase upon re-exposure to fresh 100 mM urea, indicating retained catalytic activity over the experimental timescale.

The collective dynamics are then compared in detail through the analysis of the horizontal and vertical intensity projections (Fig. 3Ci, ii) and the derived FWHM (Fig. 3Ciii), showing an immediate expansion after 20 s, when the NM reach the surface. This expansive behavior is only observed for the NM in urea, while NM in water and inactive NP in urea and water show only a slight dissipation below the surface due to passive diffusion (Fig. 3B). The horizontal projections also confirm the mirroring effect and particle sedimentation for all conditions (Fig. 3Ci). The spreading is in all cases isotropic, i.e., no significant conditionality can be observed. Importantly, the observed trends are not inferred from a single image-derived metric alone, but are consistent with the corresponding FWHM, center of mass (CoM), and top/side-view analyses.

Hence, only when observing through the side view (Fig. 3A right, Fig. 3B bottom), we can correctly assess the collective phenomena taking place: i) urea in the media causes the upwards buoyancy effect in both NM and NP as the samples in water are the only ones that sediment and ii) the expansion of the NM in urea is triggered when in contact with the air-water interface. These aforementioned aspects combined are the basis of the mechanism of collective motion taking place, highlighting the importance of both investigating motion in 3D and testing inactive particles in the presence of fuel.

**Fig. 4:**
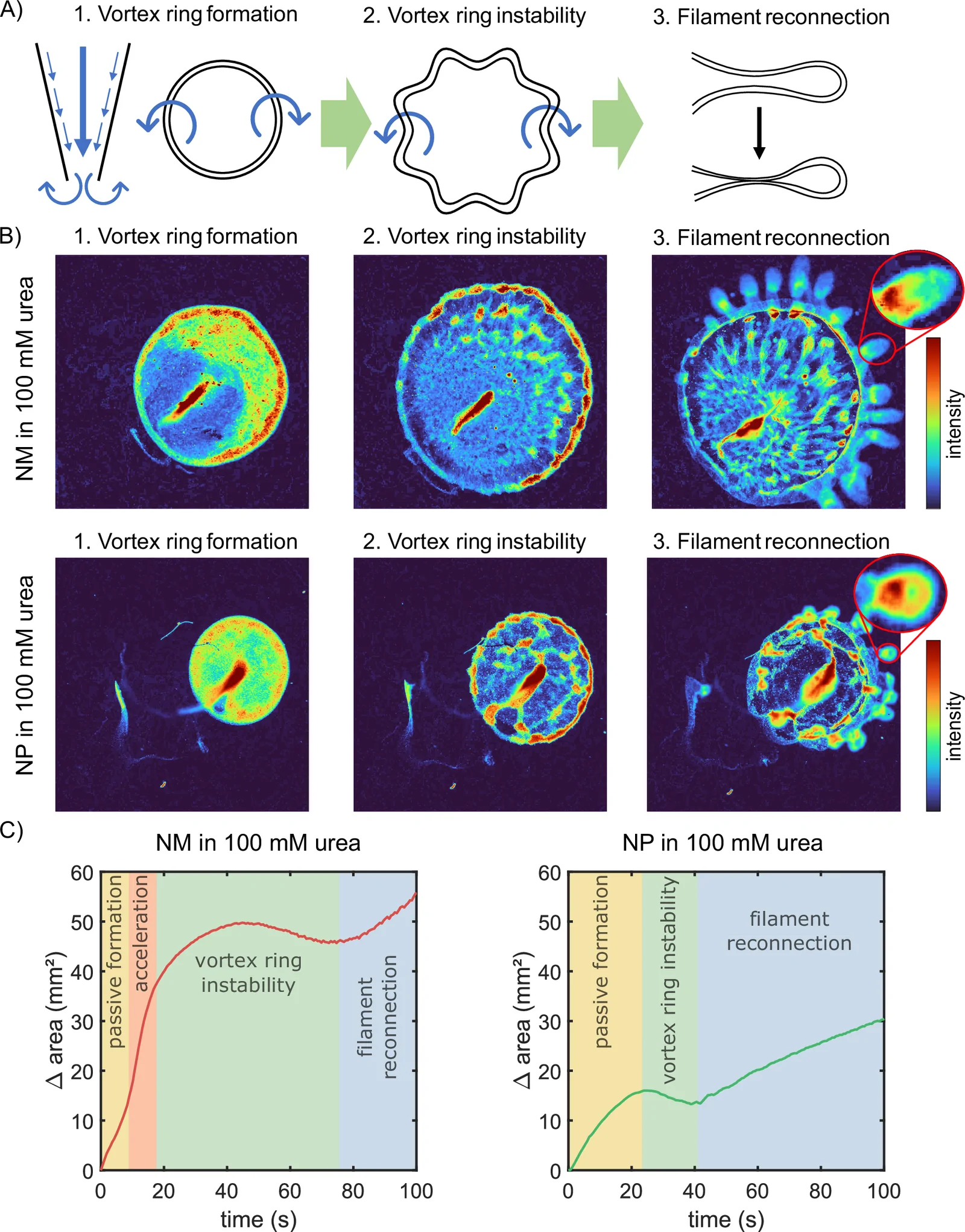
Early injection-seeded vortex-ring-like hydrodynamic redistribution of the particle swarm. **A** Three phases of vortex ring evolution: 1. Vortex ring created due to the release of enzymatic nanomotors (NM) or passive nanoparticles (NP) suspension through a pipette tip. 2. Formation of a periodic instability along the ring and 3. further looping and reconnection of the ring forming loops/secondary rings. Blue arrows represent the direction of the flow. **B** Representative snapshots of the three phases of the vortex ring for NM and NP in optimal conditions (Video 5). **C** Analysis of the area covered over time by NM and NP in optimal conditions.

Interestingly, as the cloud of NM and NP in urea evolves after injection, a ring of higher particle concentration forms in certain cases. This phenomenon can be explained within the framework of vortex ring theory, as we observe behavior analogous to that seen in fluid dynamic studies of mixing fluids with differing densities. The overall behavior originates from the interaction of two aqueous solutions, one containing urea and the other containing suspended particles. We assume that the fast release of the particle suspension from a pipette tip is the initial trigger for vortex ring formation. Because the flow is parabolic, the pipette generates shear: the fluid at the center exits faster than near the wall due to friction, causing the emerging jet to curl into a toroidal vortex as it leaves the tip (Fig. 4A). This initial stage corresponds to the first phase of vortex ring formation. As the ring propagates through the medium, it undergoes instabilities, manifesting as periodic wave structures that develop around the toroid. With increasing amplitude, these instabilities lead to the formation of loops, which can reconnect and give rise to secondary vortex rings. This sequence is consistent with the three classical phases observed in numerical simulations^73^, (i) vortex ring formation, (ii) development of instabilities, and (iii) formation of secondary rings. We interpret this structure as an early injection-seeded hydrodynamic mode that helps explain the full initial redistribution of the particle cloud, rather than as part of the central catalysis-driven swarm mechanism itself. In this sense, the vortex-ring-like behavior precedes the later stages, and its understanding is relevant to assessing the early redistribution of the injected cloud before the magnetic localization and catalysis-dependent spreading discussed in the following sections.

This dynamic behavior is observed specifically in urea-containing solutions (Fig. 4B). When the NM or NP are injected into pure water, the particles simply sediment due to the absence of buoyant support. In contrast, the presence of urea increases the density of the surrounding medium, providing sufficient buoyancy for the vortex ring to travel and dissipate through the liquid. We therefore attribute the vortex-ring-like behavior to the combination of injection-seeded hydrodynamics and urea-enabled buoyancy, rather than to catalysis itself. In the active NM condition, catalysis becomes relevant only later, once the cloud reaches the air-water interface, where it can trigger the interfacial expansion described above. For this reason, we treat the vortex-ring-like behavior as a secondary, but informative step in the overall evolution of the injected cloud, rather than as the novelty or core mechanistic claim of the work. This early hydrodynamic transport mode may still be relevant in realistic deployment scenarios where nanomotors are introduced by bolus injection or catheter-based administration, since the initially injected cloud can undergo redistribution before subsequent magnetic localization or catalytic interfacial spreading. The temporal evolution of this redistribution can also be followed directly in Video 5 and in the associated intensity-based analysis. Moreover, the catalytic activity of the NM, by reducing the surface tension through basic pH, allows for the vortex ring to expand radially over longer distances (Fig. 4B and Video 5). After the ring reaches the liquid surface, secondary rings are observed, which move downward out of the focal plane. These phenomena are probably Rayleigh-Taylor-like instabilities emerging from the mixing of fluids with different densities. Quantitative analysis of area coverage (Fig. 4C) further supports these interpretations, showing three distinct phases: passive expansion (yellow), accumulation and instability (green), and secondary ring formation (blue). Notably, NM in urea show an accelerated expansion during the initial phase, as indicated by a steeper slope (red), attributed to the catalytic expansion of the particles. This results in a significantly greater area coverage for NM compared to NP, further validating the role of enzymatic activity and interfacial fluid dynamics in shaping the observed behavior.

### Magnetic guidance of urease-magnetosome nanomotor swarm

**Fig. 5:**
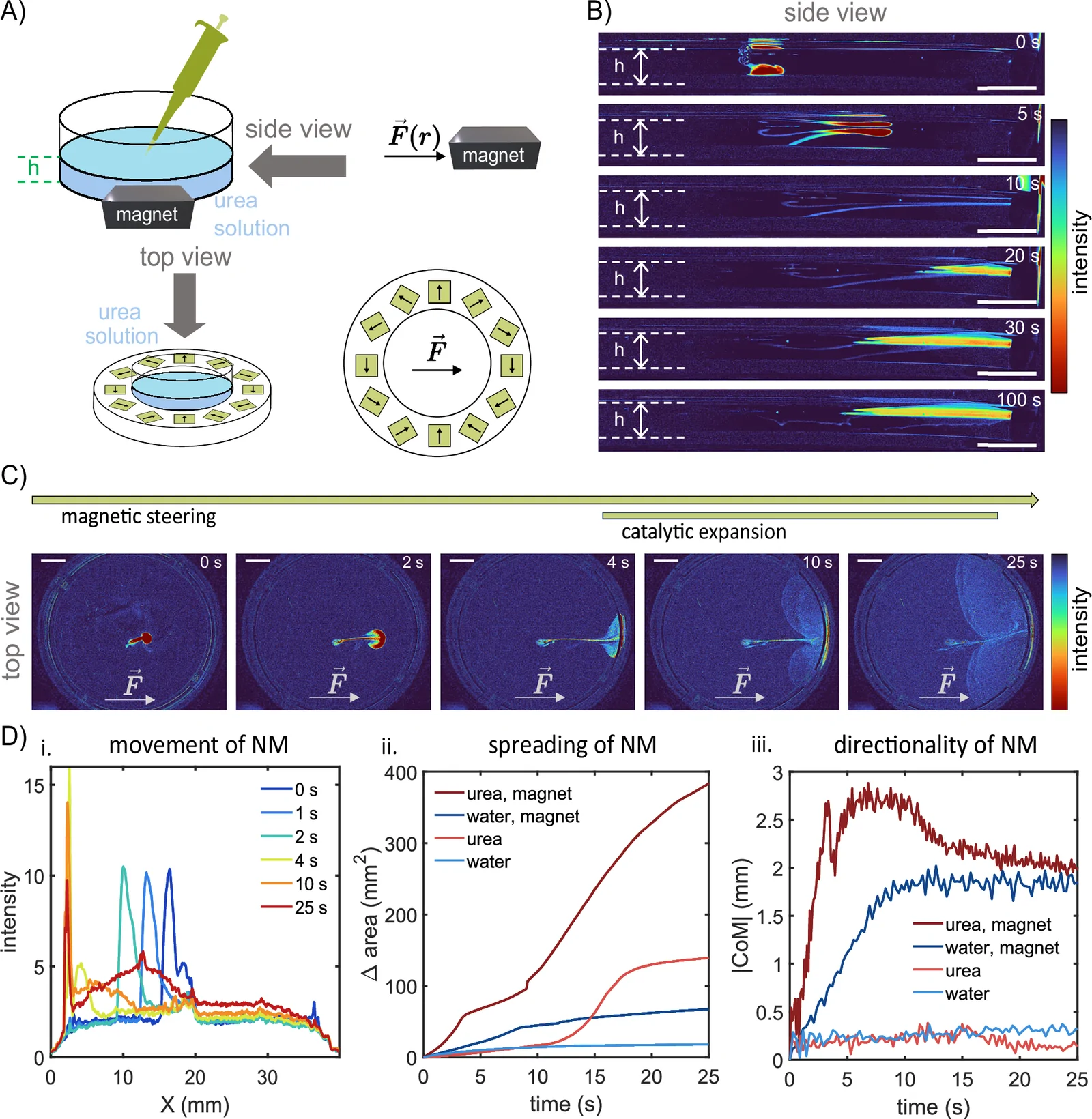
Magnetic steering of urease-magnetosome nanomotor collective dissipation. **A** Schematic of experimental setup based on a Petri dish filled with urea solution next to a permanent magnet and recorded from side view (top) and setup based on a Petri dish placed in the center of the Halbach ring for recording from top view (bottom). The force $\vec{F}(r)$ acting on the NM depends on the distance *r* to the permanent magnet. Inside the Halbach ring the force $\vec{F}$ is constant. **B** Side view snapshot of post-processed videos for different time points (0, 5, 10, 20, 30, 100 s) since addition of the NM in the 100 mM urea solution of a controlled height (h) (Video 6). **C** Top view snapshots of post-processed videos for different time points (0, 2, 4, 10, 25 s) since addition of the NM in the 100 mM urea solution (Video 6). **D** Computational analysis of the nanomotor (NM) swarming behavior as i. projections of the swarm intensities at different time points (0, 2, 4, 10, 25 s), ii. cumulative area occupied by NM and iii. directional movement based on the CoM when applying a constant magnetic force with a Halbach ring. The control condition of NM in water can be found as well in Video 6.

Finally, we examine how magnetic guidance can be combined with catalytic expansion as a two-step collective strategy. The NM cloud is first magnetically localized during the initial 20 s latency window and only then allowed to undergo catalytic expansion at the target. The optimal conditions of nanomotor concentration, urea concentration, and viscosity are used to generate the swarming behavior next to a permanent magnet (non-linear magnetic gradient field, i.e., increasing force towards the magnet) (Fig. 5A top) or inside a Halbach ring (linear magnetic gradient field, i.e., an everywhere constant force) (Fig. 5A bottom). Further description and a finite element simulation of the magnetic field generated by the Halbach ring can be found in the Supplementary Information (Section 3) and Fig. S6. The geometry of the Halbach ring does not allow for a side view observation; therefore, it is only applied for top-view observation, while the single permanent magnet is used for side-view analysis. In all settings, the magnetic force is maintained during the catalytic expansion. In Fig. 5B, we observe the NM behavior from the side view through a series of snapshots at different time points (0, 5, 10, 20, 30, and 100 s), where the NM are dragged towards the higher field strength below the air-water interface, reaching the wall of the Petri dish before 10 s. At the target location, the NM undergoes the dissipative and catalytic behavior at 20 s, as analyzed in the prior sections. Thus, in the open interface Petri-dish experiments, which constitute the core setting of this work, the main advantage is not higher velocity, but the ability to first guide and localize the swarm directionally and then expand it across a target area at the air-water interface. Hence, the concentrated cloud of particles is attracted towards a target area within the initial 20 s in order to generate the exploratory expansion only covering the targeted side of the Petri dish. Importantly, the Marangoni-driven interfacial expansion exceeds the counteracting magnetic force, allowing directional guidance of the ensemble without sacrificing local coverage once the target is reached.

We observe the same behavior from a top view inside a Halbach ring (Fig. 5C) to accurately control the specific direction of attraction and to apply a constant force without accelerating the particles' movement. The results further confirm that due to the magnetic force, the NM first reaches the wall of the Petri dish (before 4 s) and then the catalytic expansion takes place. The corresponding videos (Video 6) have been analyzed as aforementioned and the resulting intensity projections are shown in Fig. 5D. We first observe the highest intensities of NM detection moving from the center to the side of the Petri dish, and then the NM move again to the center of the Petri dish with a broader and expansive area distribution at time points of 10 s and 25 s (Fig. 5Di). This is also shown when evaluating the area covered by the NM (Fig. 5Dii), demonstrating the spreading after 10 s.

Since the spreading of the nanomotor cloud in a magnetic field is non-isotropic, the covered area is calculated by summing up pixels with intensities above a certain threshold (see Methods) and not as before by calculating the FWHM. The total area explored is 2-folded when magnetically pulling the NM or NP, which is explained by the enhanced exploration from the center to the wall of the Petri dish due to the drag of the particle cloud. The time-resolved progression from localization to redistribution is also visible in Video 6 and in the corresponding intensity projections. The displacement of the CoM is additionally used to quantify the redistribution (Fig. 5Diii). These analyses show that the NM in urea exposed to a magnetic field combines strong target localization with the widest post-target spreading behavior among the tested conditions, showing higher directionality and wider spreading than any other control condition without substrate or catalytic activity.

As a qualitative support for this magnetic-localization/catalytic-redistribution sequence, we also performed an exploratory Particle Image Velocimetry (PIV) analysis of the same image sequence as shown in Fig. 5C (Fig. S7). During the first seconds, the velocity field points predominantly toward the target in the direction of the magnetic force, consistent with magnetic localization of the NM cloud. After the catalytic response starts, the velocity field shows redistribution away from the localized region, including motion components partially opposite to the magnetic force direction, supporting catalysis-triggered interfacial spreading at the target site. Because the signal corresponds to a dense, deformable, three-dimensional particle cloud projected into two dimensions, this PIV analysis is used only as qualitative support, while the main quantitative comparison relies on covered area, CoM displacement, intensity projections, and image sequences.

**Fig. 6:**
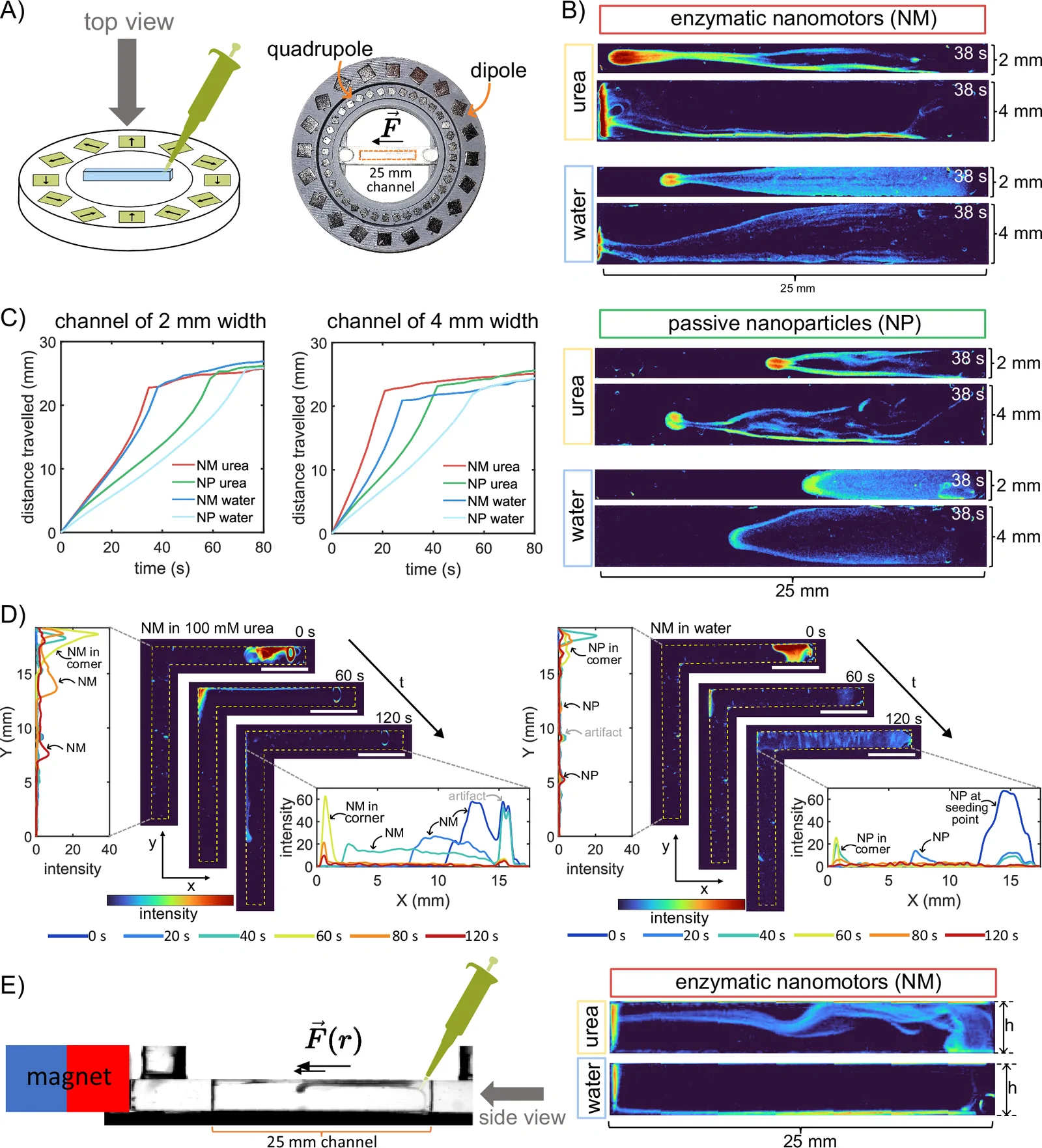
Channel confinement of the urease-magnetosome nanomotor’s swarm behavior under magnetic steering. **A** Schematic and picture of the experimental setup based on a Halbach ring around the channels of 2 mm and 4 mm width and 25 mm length generating a constant force $\vec{F}$. **B** Representative top-view snapshots of the nanoparticle cloud behaviors in both channel (2 and 4 mm of width) at the time point of 38 s for active NM and passive NP, with and without urea (Video 7). **C** Distance traveled by the front of the particle cloud through both channels (2 mm and 4 mm of width) over time for active NM and passive NP, with and without urea. **D** Magnetic steering of urease-magnetosome swarms in L-shaped channels. Representative top-view snapshots of NM with and without urea through an L-shaped channel (rotating the Halbach ring by 90^∘^ after 1 min) (Video 8). Projections of the two branches showing the amount of NM steered before and after changing the direction of the magnetic force. All scale bars are 5 mm. **E** Representative side-view snapshots of the NM cloud behavior in 2 mm channel width at the time point of 60 s with and without urea experiencing a force $\vec{F}(r)$, which depends on the distance *r* to the permanent magnet (Video 9).

Further, we test the swarm behavior while magnetically pulling the NM in confined channels. The constant force of the Halbach ring is used to drag the cloud of nanomotors in a channel of 25 m length with two alternative widths (2 and 4 mm) to study the effect of the confinement degree on the catalytic expansion from a top view (Fig. 6A). In Fig. 6B, representative snapshots of the swarming behavior of NM and NP in 0 or 100 mM urea (at optimal NM concentration and media viscosity) are shown at 38 s since addition (Video 7). Remarkably, in either width of channel, NM in urea is the fastest in reaching the opposite extreme of the channel after 39 s for a channel width of 2 mm and 25 s for a channel width of 4 mm, while NM in water display a relatively slower transport crossing the channel in 43 and 33 s for 2 and 4 mm channel width, respectively. However, there is no catalytic expansion covering the overall target area as shown in all the prior sections in open environments. This indicates that the confinement of the channel blocks the interface spreading phenomenon observed in open conditions, probably due to a deformed interface and restricted substrate/product gradients due to the lateral confinement and, hence, suppressing the Marangoni-driven expansion. Additionally, larger widths (4 mm) allow for a greater enhancement of speed in contrast to more confined widths (2 mm) in either 0 or 100 mM urea. This is explained by the increased hydrodynamic drag from the side wall to the cloud of nanomotors when the confinement is narrower due to the no-slip boundary condition at the walls: the closer a particle is to a wall, the more viscous resistance. In the case of NP, all scenarios display 40–46% slower motion than their NM counterparts (Fig. 6C), probably because the more negative charge of the NM provided by urease may allow for electrostatic repulsion with the wall and less hydrodynamic drag. Hence, when comparing the conditions of NP or NM, the following conclusion is reached for larger channel widths: the combined effects of urea-driven buoyancy and the more negative particle charge promote faster magnetic steering.

To better understand the magnetic swarm behavior in more complex environments, the channels of 25 mm length and 2 mm width are extended in an L-shape with both branches having the same length. In Fig. 6D, multiple snapshots are presented showing the guidance in L-shaped channels for different time points (0, 60, and 120 s) for NM in 0 or 100 mM urea (Video 8). The NM are first pulled using the Hallbach ring to reach the end of the channel to then change the pulling direction by rotating the Halbach ring by 90 ^∘^ and observe the behavior from a top view (the rest of conditions are maintained as before). As observed in previous experiments, the snapshots and the analysis show that NM in urea travel faster towards the opposite extreme of a linear channel.

Interestingly, after crossing the first linear section of the channel and making the 90 ^∘^ turn of the magnetic field to guide the NM through the second section of the L-shaped channel, a considerable quantity of NM in water is stuck in the path of the first section, unable to follow the L-shaped channel. Hence, the bottom surface and side wall of the channel is retaining part of the NM on its path. Hence, the NM in urea reaches the end of the L-shaped channel in significantly higher concentrations and without much observable NM left behind due to the buoyancy effect of urea, avoiding sedimentation and allowing a faster and more cohesive motion of the swarm. Consistent with the prior motion experiments in confinement, no catalytic expansion is observed, stressing the hindering effect of confinement on the spreading phenomenon at the air-water interface.

To elucidate this enhancement of speed due to urea addition, the NM are magnetically steered with a permanent magnet through a channel of 25 mm of length and 2 mm of width and recorded from a side view. As observed in Fig. 6E, the side-view snapshots show that NM in water sediment and are transported across the bottom of the channel, while the NM in urea cover all the height of the channel. This is due to the buoyancy effect of urea on the NM swarm allowing them to separate from the bottom surface and navigate more cohesively, hence, faster to the opposite extreme of the channel (Video 9).

Consistent with the prior motion experiments in confinement, no catalytic interfacial expansion is observed, confirming that the free-surface mode is suppressed. Thus, under confinement, the relevant outcome is not the full swarm functionality seen in open environments, but rather a more limited transport behavior governed by reduced bottom contact and more cohesive magnetic motion. In this sense, the confinement results serve primarily to define the conditions under which the complete combined swarm behavior is hindered, rather than to demonstrate a strong performance advantage in confined geometries.

Collective motion enables exploration of larger areas, greater adaptability in intricate environments, and higher on-target agent concentration. Here, we establish a mechanistic proof-of-principle for a two-step magneto-enzymatic collective strategy: magnetic localization of urease-powered, magnetosome-based nanomotors followed by catalysis-triggered Marangoni expansion. By resolving the behavior from both top and side views and by comparing active NM with inactive NP in urea, we clarify the mechanism of catalytic expansion, an aspect often overlooked. We conclude that urea establishes vertical concentration gradients that promote upward accumulation of both NM and NP toward the air-water interface, whereas only active NM undergo abrupt spreading. This catalytic expansion is enabled by ureolysis locally basifying the interface and creating surface-tension gradients that drive outward Marangoni flows. This Marangoni-driven spreading is parameter-sensitive: i) the magnetosome or urea level is too low to produce a sharp interfacial pH change, ii) excess particle loading promotes dipole-dipole chaining and sedimentation, iii) higher viscosity damps the flow by increasing effective interfacial viscosity/elasticity and reducing surface-tension gradients, and iv) confinement further suppresses spreading, likely by flattening surface-tension gradients. Under a magnetic gradient field, we steer the swarm and time the catalytic expansion at the target: the latency before expansion is leveraged to accumulate NM in open settings and then achieve directional coverage. Notably, interfacial expansion ultimately overpowers the opposing magnetic force. Together, these results establish both a distinct catalytic collective mechanism and a magnetic-catalytic swarm strategy in which directional localization can be combined with expansive target-area coverage. More broadly, we define the governing balances and threshold conditions under which this combined swarm behavior is enabled, and provide a fundamental basis for future environmental and biomedical nanomotor applications.

## Methods

### Chemicals

1 × 4-(2-hydroxyethyl)-1-piperazineethanesulfonic acid (HEPES) (Thermo Fisher Scientific cat. no. 15630080), 2,2’,2”,2”’-(Ethane-1,2-diyldinitrilo)tetraacetic acid (EDTA) (Sigma-Aldrich cat. no. 798681), glycerol (Sigma-Aldrich cat. no. G7893), sodium chloride (NaCl) (Sigma-Aldrich cat. no. S9888), phosphate-buffered saline (PBS) (Thermo Fisher Scientific cat. no. 70011-036), urease from *Canavalia ensiformis* (Jack bean) (Sigma-Aldrich cat. no. U4002), urea (Sigma-Aldrich cat. no. U5128), Pierce™ BCA Protein Assay Kit (Thermo Fisher cat. no. 23227), Urease Activity Assay Kit (Sigma-Aldrich cat. no. MAK120),

### Instruments

TEM images were captured by a Tecnai G2 BioTWIN (FEI company). The zeta potential (*ζ*-potential) measurements were performed with a Zetasizer Nano S from Malvern Panalytical. The absorbance measurements were done with the Benchmark Plus Microplate reader from Bio-Rad. The diffusion coefficient measurements were performed using a Möbius from Wyatt Technology. The optical videos of urease nanomotors were recorded using the camera (Hamamatsu Digital Camera C11440) of an inverted optical microscope (Leica DMi8). The large field of view and the side-view videos were recorded with a digital camera with a 35 mm objective (Thorlabs, Inc, Newton, New Jersey). The stereolithographic printing of the channels was conducted with a Form2 3D-printer (Formlabs, Somerville, Massachusetts). The magnetic flux density was measured with a magnetometer (Wuntronic GmbH, Munich, Germany).

### Magnetotactic bacteria cultivation and magnetosome purification

The magnetotactic bacteria strain *Magnetospirillum magneticum* AMB-1 was produced at the Biomasse-Fermentation platform of the Marseilles IMM Institute. AMB-1 was anaerobically grown in Sartorius BIOSTAT-C30 (30 L) bioreactors until late exponential phase. Bacterial cells were harvested by centrifugation for 20 min at 4000 g and stored at  −20 ^∘^C until use. Purified magnetosome suspensions were prepared. Each 4 g bacterial cell pellets were resuspended in 30 mL of Buffer 1 (20 mM HEPES, 1 mM 2,2’,2”,2”’-(Ethane-1,2-diyldinitrilo)tetraacetic acid (EDTA), 8% glycerol, 0.9% NaCl, pH 7.5) with 20 μL of anti protease inhibitor cocktail (Sigma P8849). Bacterial cells were disrupted three times with a French press (1200 psi-8.27 × 106 Pa). Magnetosomes were magnetically purified using a MACSiMAG™ separator (MiltenyiBiotec) and washed five times in 10 mL of Buffer 1 and 5 times in 10 mL of Buffer 2 (20 mM HEPES, 8% glycerol, 0.9% NaCl, pH 7.5). Magnetosomes were finally resuspended in 200 μL of Buffer 3 (20 mM HEPES, 8% glycerol, pH 7.5), flash-frozen in liquid nitrogen and stored at  −80 ^∘^C.

Magnetosome suspensions were quantified according to their iron content. The iron concentration was determined by Inductively Coupled Plasma Atomic Emission Spectroscopy (ICP-AES) after mineralization in the presence of concentrated nitric acid and overnight incubation at 80 ^∘^C

### Transmission Electron Microscopy (TEM) micrographs of magnetosomes

Transmission electron microscopy (TEM) (Fig. 1B) was performed on magnetosomes deposited onto 200-mesh formvar carbon-coated copper grids and washed three times with MilliQ water. The images were collected using a Tecnai G2 BioTWIN (FEI Company) electron microscope equipped with a charged-coupled device (CCD) camera (Megaview III, Olympus Soft imaging Solutions GmbH) using an accelerating voltage of 100 kV. For crystal size measurements, TEM images were analyzed using the Fiji software. The mean and Standard deviation was determined.

### Fabrication of urease-powered magnetosome nanomotors

To fabricate urease nanomotors (Fig. 1A), magnetosomes were washed three times with 1 × phosphate-buffered saline (PBS) (Thermo Fischer Scientific cat. no. 70011-036). Then, the magnetosomes were suspended in 1 × PBS containing 3 mg mL^−1^ of urease from *Canavalia ensiformis* (Jack bean) (Sigma-Aldrich cat. no. U4002). The solution was left overnight and washed 3 times with 1 × PBS. The supernatants discarded in this process were used for the total protein quantification (Fig. 1C). Then, the solution of urease magnetosomes in 1 × PBS is divided in aliquots and stored at 4 ^∘^C to be used the same day. The functional retention of the non-covalently adsorbed urease after repeated washing and catalytic operation was further evaluated through the pH recovery assay described below.

### Zeta potential measurements

The zeta potential of the magnetosomes (Fig. 1C) were measured in each step of the functionalization process through DLS. The Zetasizer Nano S from Malvern Panalytical was used for zeta potential characterization. For zeta potential measurements, the samples were measured for a minimum of six times per condition with a scattering angle of 173^∘^ and using the Henry equation. All the results are presented as mean  ± standard error of the mean.

### Total protein quantification

The presence of enzyme attached on the surface of magnetosomes was confirmed indirectly through a total protein quantification (Fig. 1C) of the supernatants discarded on the 1x PBS washes of the functionalization process (Fig. 1A). The protocols Preparation of Standards and Working Reagents and Microplate Procedure (Sample to WR ratio = 1:8) were followed to perform the total protein quantification as specified in the document of Instructions of the Pierce™ BCA Protein Assay Kit (Thermo Fisher cat. no. 23227). By these means, the protein quantified in the supernatants discarded after functionalization was subtracted to the protein quantity contained in the enzyme powder to obtain the mass and percentage of urease attached. All the results are presented as mean  ± standard error of the mean.

### Urease enzymatic activity

The enzymatic activity of urease adsorbed on magnetosomes (Fig. 1E) was evaluated for all different urea concentrations (25, 50, 100, 200, 400, 800 mmolL^−1^) using the Urease Activity Assay Kit (Sigma-Aldrich cat. no. MAK120) based on the Berthelot method.6 The process followed is detailed in the Technical Bulletin of the Urease Activity Assay Kit. It works through the ammonia generated (Equation (1)) obtained in the catalysis of urea (Sigma-Aldrich cat. no. U5128) by urease and monitoring the absorbance at 670 nm^74^. The enzymatic activity is investigated for 3 min in the different conditions, by incubating the urease nanomotors in the different conditions with the urea provided in the kit. All the results are presented as mean  ± standard error of the mean.

### Analysis of nanomotor diffusion

The Möbius from Wyatt Technology was used to analyze the electrophoretic mobility through dynamic light scattering (DLS) and extract the diffusion coefficient of the urease nanomotors (Fig. 1D). Active motion of the nanomotors was studied as a function of different concentrations of urea (25, 50, 100, 200, 400, 800 mmolL^−1^). The hydrodynamic radius is correlated with the diffusion coefficient according to the following Einstein-Stokes equation

2 $$D=\frac{{\mathrm{k}}_{{\rm{B}}}T}{6\pi \eta {r}_{{\rm{h}}}}$$

where k_B_ is the Boltzmann constant, *T* is the absolute temperature, *η* is the solvent viscosity and *r*_h_ is the hydrodynamic radius of the diffusing particle. The electrophoretic mobility was studied using an acquisition time of 5 s, with a laser of 532 nm wavelength and a detector angle of 163.5 ^∘^. For each condition, the diffusion coefficient was calculated the average of 14 − 18 acquisitions obtained directly from the analysis of the scattering data on the Dynamics software. All the results are presented as mean  ± standard error of the mean.

### Optical video recording

For recording the videos from top view and side-view a camera was used with a frame rate of 10 fps, a resolution of 1024 × 1280 and a recording time of 2 min. Only for the recording of the swarming behavior for different particle concentrations the videos were recorded using the camera (Hamamatsu Digital Camera C11440) of an inverted optical microscope (Leica DMi8) by using a 1.25 × water immersion objective. The videos were recorded at 25 fps and a resolution of 2048 × 2048pixels. For all experiments a Petri dish (area of 9.2 cm^2^) was used, adding 5 μL of nanomotor solution in 3 mL solution (with or without urea). To add the nanomotors or particles, the pipette tip is immersed at half the height between the Petri dish bottom and the air-water interface.

### Computational analysis of collective motion

We analyzed the recorded videos by using a self-written Matlab program. First, the videos were compressed to 300 frames, then the background has been subtracted by using one of the first empty frames as background. The gray scale has been inverted and a colormap was applied, i.e. the colormap shown in the figures and videos in the SI represents the light intensity of the original videos. This intensity is used here as a relative descriptor of the evolving particle distribution, but it is not interpreted as an absolute particle concentration because a quantitative conversion between light intensity and particle number cannot be established under the present imaging conditions. To improve robustness against moderate illumination variations, a background correction step is applied before analysis. In addition, the coverage-area metric is computed after binarization above a fixed threshold, which makes the readout relatively insensitive to illumination differences within the non-saturated, non-underexposed regime used in the present experiments. The projections in horizontal and vertical direction have been calculated, by summing up and averaging the pixel intensities along x- and y-direction on the top-view videos and x- and z-direction on the side-view videos, respectively. The projections in x and y show the spreading of the NM swarm, while the projections on the z-axis allows for the investigation of the buoyancy of the swarm, whether the particles sediment or move towards the surface. The projection of one row or column reads:

3 $$\bar{I}=\frac{1}{{N}_{i}}\mathop{\sum }\limits_{n=1}^{N}{I}_{n}$$

with *N*_*i*_ either being the number of pixels in a row or in a column and *I*_*n*_ the intensity of pixel *n*. To quantify the distribution of particles the FWHM of the vertical projections have been calculated i.e., the width of the projections was measured by finding the corresponding x-value of the half of the distribution’s maximum intensity value. The FWHM quantifies the spreading of the swarms and is directly correlated to the area occupied by the particles since they spread isotropically. When applying a magnetic field, the distribution is not uniformly anymore, but is showing a direction. Therefore, the cumulative area occupied by particles has been derived from the top-view videos. For this, a threshold of 2% has been applied to obtain binary images. All pixels with an intensity value above this threshold are considered to be particles. The area of the *k*th frame reads

4 $${A}_{k}=\mathop{\sum }\limits_{n=1}^{N}{I}_{k,n}^{\,\mathrm{bi}\,}$$

with *N* being the total number of pixel in the frame and the binary image calculated as follows

5 $${I}_{n,k}^{\,\mathrm{bi}\,}=\left\{\begin{array}{l}1\,\mathrm{if}\,\,{I}_{n,k} > 0.02\cdot \,{I}_{\max }\quad \\ 1\,\mathrm{if}\,\,{I}_{n,k-1} > 0.02\cdot \,{I}_{\max }\quad \\ 0\,\mathrm{else}\,\quad \end{array}\right.$$

accounting also the pixel in the calculated frame as area occupied by particles, which exceeded the threshold in any frame before. To quantify the overall directionality of the movement of particles, the CoM of a frame has been calculated as follows

6 $${{\bf{r}}}_{{\rm{CoM}}}(k)=\frac{\mathop{\sum }\nolimits_{n=1}^{N}{I}_{n,k}{{\bf{r}}}_{n}}{\mathop{\sum }\nolimits_{n=1}^{N}{I}_{n,k}}.$$

### Characterization of pH during swarm behavior and retained activity after recovery post-swarm

For pH characterization, the same media compositions used in the swarm experiments were prepared and monitored over time under catalytic and control conditions. The pH evolution was measured using a GLP21 pH meter equipped with an Orion-Ross pH microelectrode. Urease-functionalized magnetosome nanomotors were tested by adding 5 μL of nanomotor suspension into 2 mL of 100 mM urea solution and monitoring the pH evolution over time (from 1000 to 1800 s). As controls, the pH evolution of 100 mM urea solution alone and of magnetosomes in water was also recorded for a short time (from 60 to 120 s). To assess retained catalytic activity after recovery, the nanomotors were centrifuged for 4 min at 6000 rpm after the initial swarm experiment, the supernatant was removed while leaving less than 50 μL of residual suspension, and the particles were washed ×3 times with Milli-Q water. The recovered particles were then added again to a fresh 2 mL solution of 100 mM urea, and the pH evolution was monitored over time. The first pH measurement provides a functional readout of urease activity under swarm-relevant conditions, while the second measurement after recovery evaluates whether catalytic activity is retained after washing and prior operation.

### Particle image velocimetry analysis

Exploratory PIV analysis was performed using the MATLAB-based PIVlab package. The video used for the analysis consisted of 217 frames and corresponded to the magnetic steering experiment shown in Fig. 5C of the main manuscript. First, the background was subtracted from the video, and a circular region of interest was selected to retain only the area of the Petri dish. The grayscale intensities were then inverted so that the NM swarm appeared bright on a dark background. The resulting video was imported into PIVlab. Five frames corresponding to the time points 0, 2, 4, 10, and 25 s, matching those shown in Fig. 5C, were selected for analysis. The velocity fields were calculated using the Multipass FFT window deformation algorithm, with an interrogation area of 92 px and a step size of 46 px in the first pass, followed by an interrogation area of 46 px and a step size of 23 px in the second pass. A Gauss 2 × 3-point sub-pixel estimator was used, and the correlation robustness was set to high. Because the recorded signal corresponds to a dense and deformable three-dimensional particle cloud projected into two dimensions rather than to passive tracer particles, the resulting PIV fields were used only as qualitative support for the magnetic-localization and catalytic-redistribution sequence, not as the primary quantitative analysis.

### Additive manufacturing of channels

The CAD model of the channels has been designed by using 3D builder (Microsoft, Redmond, Washington) and 3D-printed by stereolithography technique (Form2, Formlabs, Somerville, Massachusetts) using the Clear resin and applying a layer thickness of 25 μm (Fig. 6). The printed objects have been washed with Isopropanol and cured under UV-light for 20 min. Afterwards the supporting structures have been removed, and the channels have been sanded thoroughly to polish the surface. Then they were coated with an impregnating agent (Nanoseal 180W+, Jeln Imprägnierung GmbH, Schwalmtal, Germany) to obtain transparent channels.

### Halbach ring for magnetic guidance

For the application of a linear gradient field, two Halbach rings have been designed, one as a dipole and one as a quadrupole (Figs. 5, 6 and S6). The superposition of the dipole and the quadrupole field is a linear gradient field, which allow the application of a constant force on magnetic nanoparticles, facilitating a magnetic moment *m*^75^. Assuming that the applied magnetic field strength is sufficiently high to saturate the magnetization of the particles, the force acting on a single particle can be given as

7 $${\bf{F}}={m}_{{\rm{s}}}\nabla \left\vert {\bf{B}}\right\vert$$

The Halbach rings have been designed, such that they can be equipped with commercially available NdFeB magnets and that they fit into the microscope holder. The number of magnets has been calculated according to ^76,77^. A numeric simulation has been performed (FEMM 4.2, Magnetics). The holder for the magnets has been designed by using 3D Autocad (Autodesk, Inc., San Francisco, California) and 3D-printed (Form2) by using the gray resin. The NdFeB N45 magnets of the dipole have a size of 6 × 6 × 6 mm^2^ and the ones of the quadrupole 3 × 3 × 3 mm^2^ (Superimanes S.L, Sevilla, Spain). The calculated and simulated gradient strength is 0.5 T/m, which could be also confirmed by measuring the flux density with a magnetometer along the ring opening. The inner ring opening is 50 mm, able to accommodate a Petri dish of 35 mm inner diameter with some spacing to account for field inhomogeneities close to the magnetic rings.

## Supplementary information

supplementary information

Description of Additional Supplementary Files

videos

Transparent Peer Review file

## Data Availability

The measurement data generated in this study have been deposited in the figshare database [https://doi.org/10.6084/m9.figshare.32592318]. Videos on which the analysis is based on can be found in the Supplementary Information.

## Code availability

The Matlab code for analyzing the videos provided in the Supplementary Information can be requested from the corresponding author.
