# Supplementary material for: Synergistic magneto-enzymatic propulsion enables multimodal nanomotor swarms
Source: Nat Commun. 2026 Aug 11;17:9660. doi: 10.1038/s41467-026-76053-x (PMC13558676; doi:10.1038/s41467-026-76053-x)
Supplement: Supplementary file 1 [file 41467_2026_76053_MOESM1_ESM.pdf]

Synergistic Magneto-Enzymatic Propulsion Enables Multimodal Nanomotor Swarms
-
Supplementary Information

Anna C. Bakenecker[1,2,*], Xavier Arqué[1,3,4,*], Xander Peetroons[1], Paul E. D. Soto Rodriguez[5,6], Sandra Prévéral[5], Damien Faivre[5], and Samuel Sánchez[1,7]

[*]contributed equally
[1]Institute for Bioengineering of Catalonia (IBEC), Barcelona Institute of Science and Technology (BIST), Baldiri i Reixac 10-12, 08028 Barcelona Spain
[2]Medical Engineering, Department of Electrical Engineering and Infromation Technology, Technical University of Darmstadt, Merckstr. 25, 64283 Darmstadt, Germany
[3]Gulliver UMR CNRS 7083, ESPCI ParisTech, PSL Research University, 75005, Paris, France
[4]Faculty of Pharmacy and Food Sciences, University of Barcelona, Av. Joan XXIII 27-31, 08028, Barcelona, Spain
[5]Aix-Marseille University, CNRS, CEA, BIAM, Saint-Paul-lez-Durance, 13115 France
[6]Instituto de estudios avanzados IUDEA, Departamento de Física, Universidad de La Laguna C/Astrofísico Francisco Sánchez, s/n.E-38203, Tenerife, Spain
[7]Department of Physics, Faculty of Science and Technology, University of Latvia, Jelgavas iela 3, 1004 Riga, Latvia
[8]Catalan Institute for Research and Advanced Studies (ICREA), Passeig de Lluís Companys 23, 08010 Barcelona, Spain

1 Optimization of swarming conditions for urease-magnetosome nanomotors

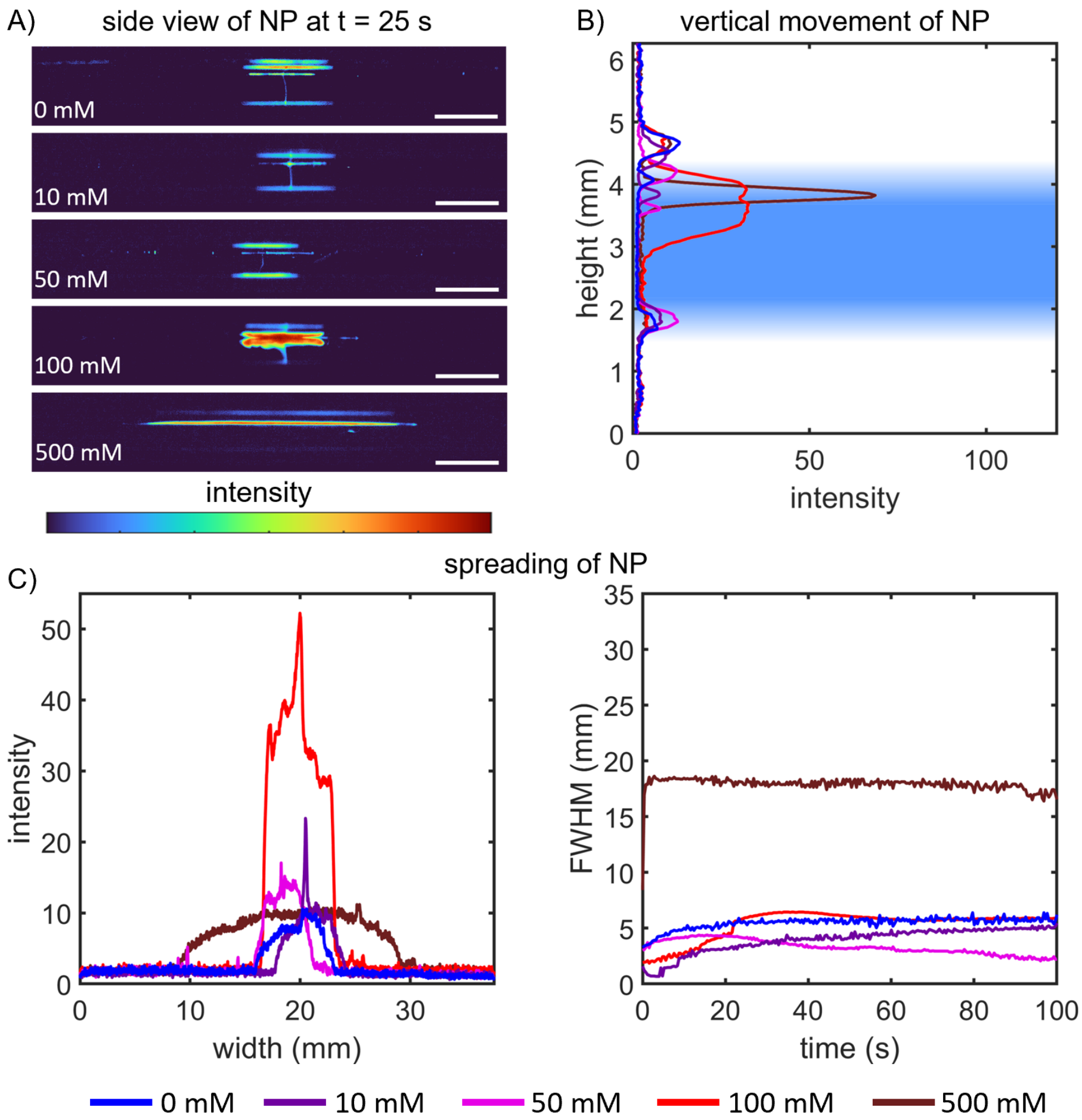

Figure S 1: Motion behavior of passive nanoparticles (NP) in different urea concentration for comparison with the behavior of active nanomotors (NM) shown in Figure 2 main manuscript. A) Snapshots of the videos at 25 s are shown for different urea concentrations (Video 1). B) The vertical movement behavior is analyzed through horizontal projections. C) The spreading of the passive NP is shown by vertical projections as well as their full width half maximum evaluation. Note: At 500 mM urea, passive NP reach the interface in a broadly redistributed state; this behavior does not correspond to the evolving catalytic expansion front observed for active NM.

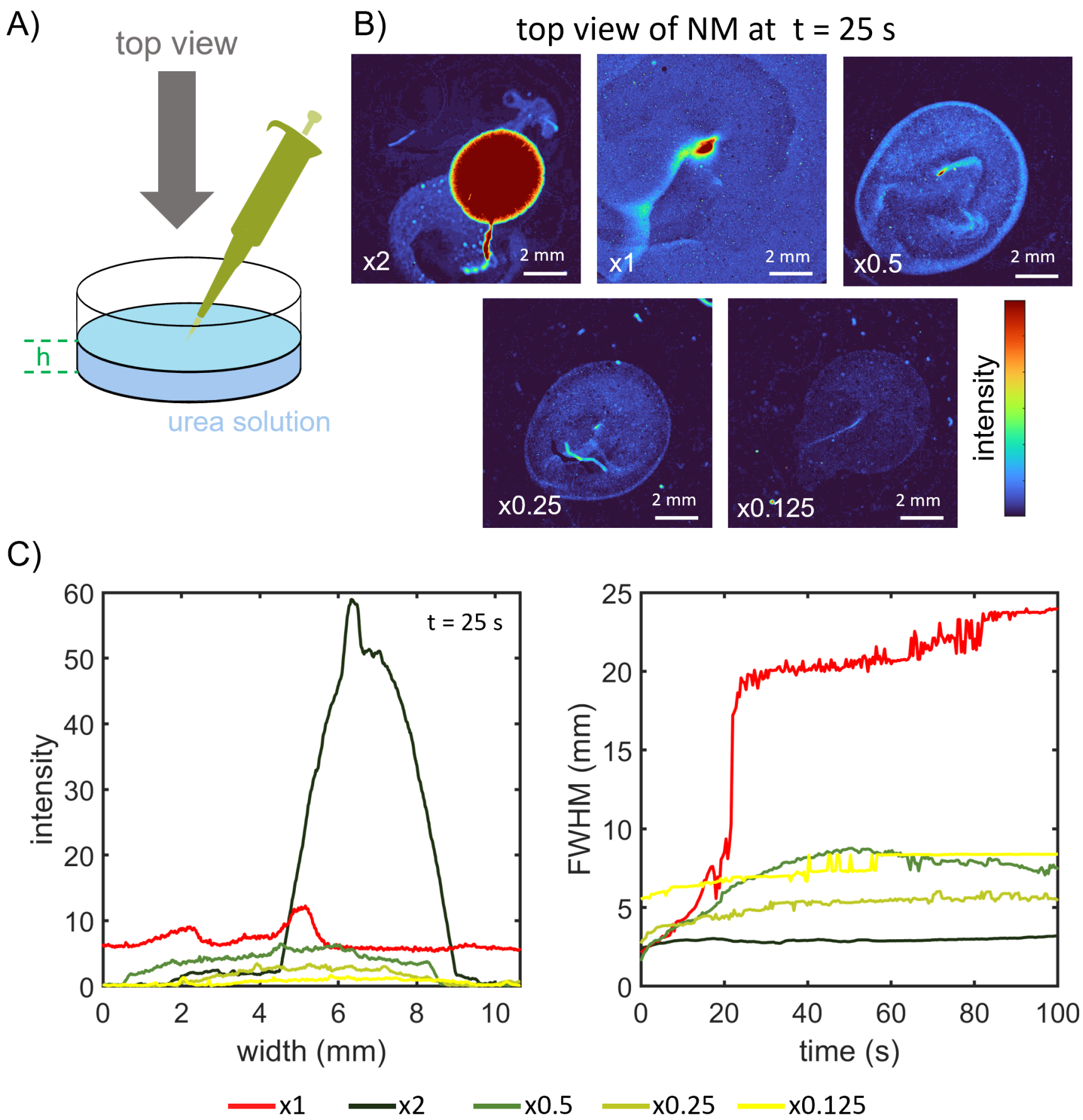

Figure S 2: Swarm motion behavior at different nanomotor (NM) concentrations. A) The NM are pipeted into a Petri dish and videos are recorded from a top view. B) NM swarms at different nanomotor concentrations ($\times 2$, $\times 1$, $\times 0.5$, $\times 0.25$ and $\times 0.125$ relative nanomotor concentration) in 100 mM urea. Representative snapshots at $t = 25\,\mathrm{s}$ from addition of swarms for different nanomotor concentrations (Video 2). C) Intensity projection for different nanomotor concentrations at 25 s from seeding and quantification of the dissipation through FWHM over time of the vertical intensity projections.

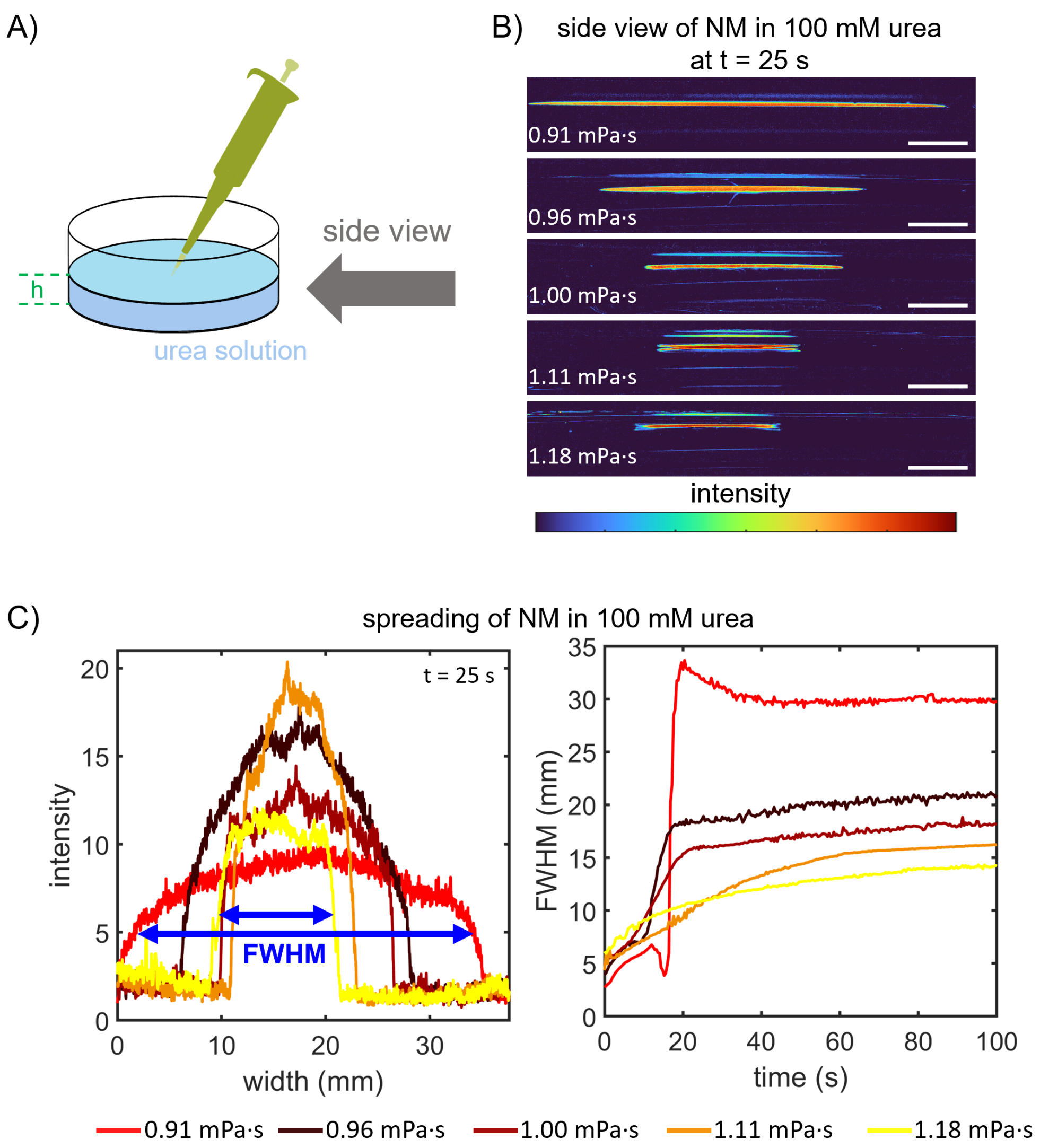

Figure S 3: Nanomotor (NM) swarm motion behavior in different viscosities of the media (0.91, 0.95, 1, 1.11, 1.19 $\mathrm{mPa \cdot s}$). The viscosity has been calculated for different glycerol-water mixtures according to [1] A) The NM are seeded into the Petri dish filled with urea containing water of 100 mM with different concentration of glycerol changing the viscosity of the medium. The lowest viscosity refers to no glycerol added. B) Representative snapshots at $t = 25\,\mathrm{s}$ from addition of NM swarms (Video 3). Scale bar 5 mm. C) Vertical intensity projections of the side view showing the spreading of the NM in horizontal direction at $t = 25\,\mathrm{s}$ for different viscosities. Quantification of the dissipation through full width half maximum over time of the vertical intensity projections of NM dissipation.

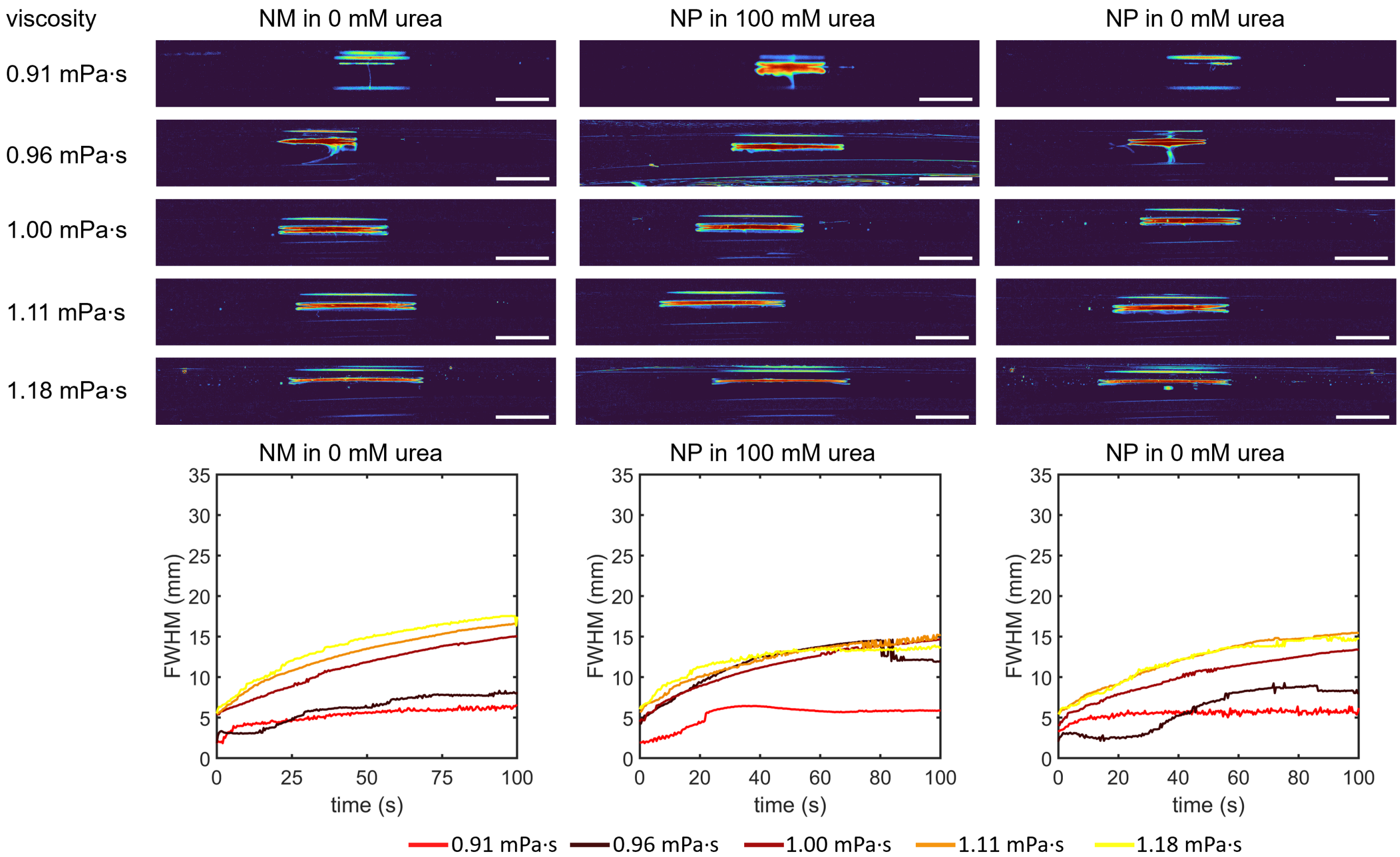

Figure S 4: Control for the motion behavior in different viscosities of the media (0.91, 0.95, 1, 1.11, 1.19 $mPa \cdot s$). The dissipation of active nanomotors (NM) without fuel as well as passive nanoparticles (NP) in 100 mM urea and without fuel are shown. Snapshots correspond to video 3 in supplementary information. The lowest viscosity refers to no glycerol added. Representative snapshots at $t = 25\,s$ are shown as well as the full width half maximum evaluation of the videos over time. Scale bar 5 mm.

2 pH characterization of urease-functionalized magnetosome nanomotors

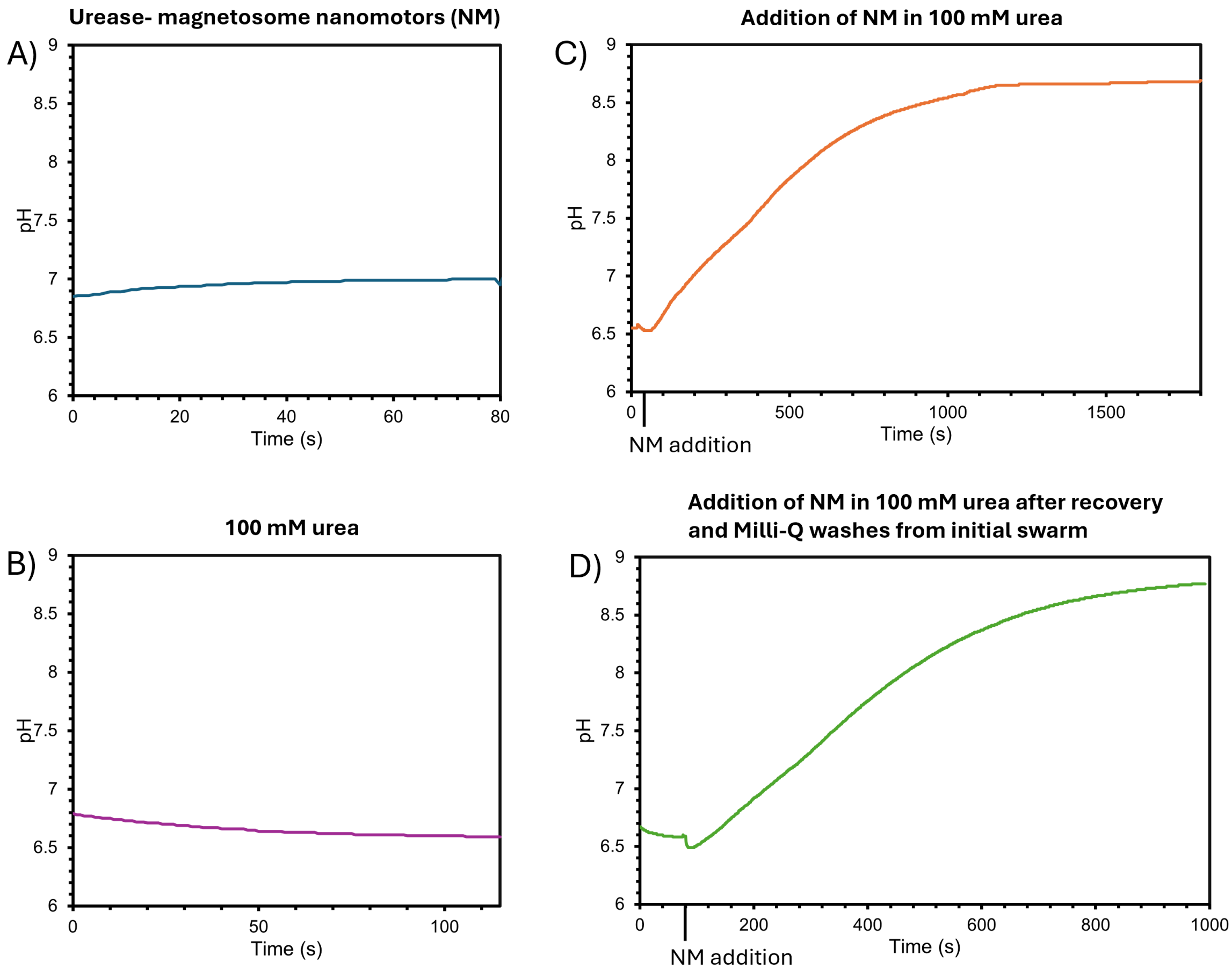

Figure S 5: pH characterization of urease-functionalized magnetosome nanomotors (NM) and retained catalytic activity after recovery. Time evolution of pH for control and catalytic conditions. A) Magnetosomes in water over time. B) 100 mM urea solution over time. C) Urease-functionalized magnetosome nanomotors added to 100 mM urea solution producing a clear pH increase, supporting ureolysis-driven alkalinization under swarm-relevant conditions. D) After the swarm experiment, the nanomotors were recovered by centrifugation, washed 3 times with Milli-Q, and re-exposed to fresh 100 mM urea solution. The recovered nanomotors still induce a clear pH increase, indicating retained catalytic activity after swarm operation and recovery.

3 Magnetic guidance

To apply a constant force, a Halbach ring consisting of a Halbach dipole and a quadrupole has been built, which provide a magnetic gradient field with constant slope (see Figure S6). Since the force reads $\vec{F} = V\nabla(m \cdot \vec{B})$ and we assume that the dipole magnetic field is sufficiently strong to magnetize the particles, so that m is the saturation magnetic moment and V the volume of the magnetic core. The dimensions of the rings have been designed such, that they can be placed into the microscope and are able to accommodate a Petri dish with a diameter of 3 cm. Because of field inhomogeneities becoming more significant towards the edges of the rings, we accounted for a safety margin of 5 mm between the dish and the ring. NdFeB magnets of grade N45 were purchased from Superimanes, Sevilla, Spain. 20 magnets cubic shaped magnets of $6 \times 6{\times}6\,\text{mm}^3$ were used to build the outer dipole ring and 36 magnets with a size of $3 \times 3{\times}3\,\text{mm}^3$ for the inner quadrupole. The magnets have been assembled into a 3D printed mold (Form2, Formlabs). Numeric simulations have been performed by using FEMM 4.2. Since this program only allows for 2D simulations, it does not provide information on the field inhomogeneities resulting from the length of the magnets. To obtain realistic values for the field strength a factor was calculated to account for the length of the magnets according to [2]. From simulations we obtain a gradient strength of 0.5 T/m.

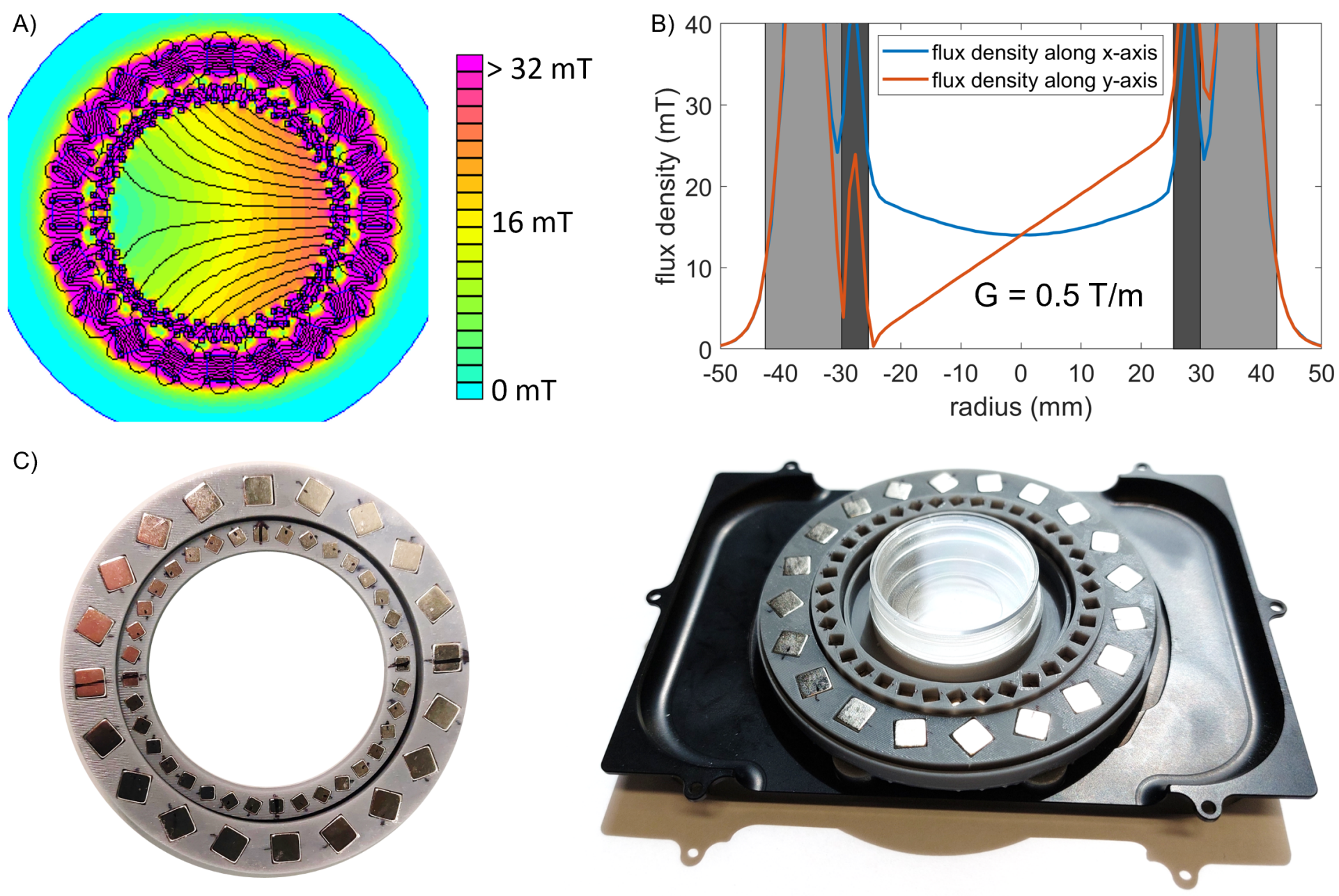

Figure S 6: The Halbach ring applies a force to the NM and NP. A) The 2D numeric simulation shows a gradient field inside the rings with higher intensities towards the left side. B) The line evaluation of the numeric simulation shows an almost linear gradient of about 0.5 T/m in one direction. The field strength goes from about 5 mT to 25 mT within the region of the Petri dish (diameter 3 cm). C) The assembled Halbach rings, which are able to accommodate a Petri dish and are manually rotatable to change the direction of the applied force.

4 PIV analysis

Particle image velocimetry (PIV) was performed using the MATLAB-based PIVlab package. The video used for the analysis consisted of 217 frames and corresponded to the magnetic steering experiment shown in Figure 5C of the main manuscript. First, the background was subtracted from the video, and a circular region of interest was selected to retain only the area of the Petri dish. The grayscale intensities were then inverted so that the NM swarm appeared bright on a dark background. The resulting video was imported into PIVlab.

Five frames corresponding to the time points 0, 2, 4, 10, and 25 s, matching those shown in Figure 5C, were selected for analysis. The velocity fields were calculated using the Multipass FFT window deformation algorithm, with an interrogation area of 92 px and a step size of 46 px in the first pass, followed by an interrogation area of 46 px and a step size of 23 px in the second pass. A Gauss 2×3-point sub-pixel estimator was used, and the correlation robustness was set to high.

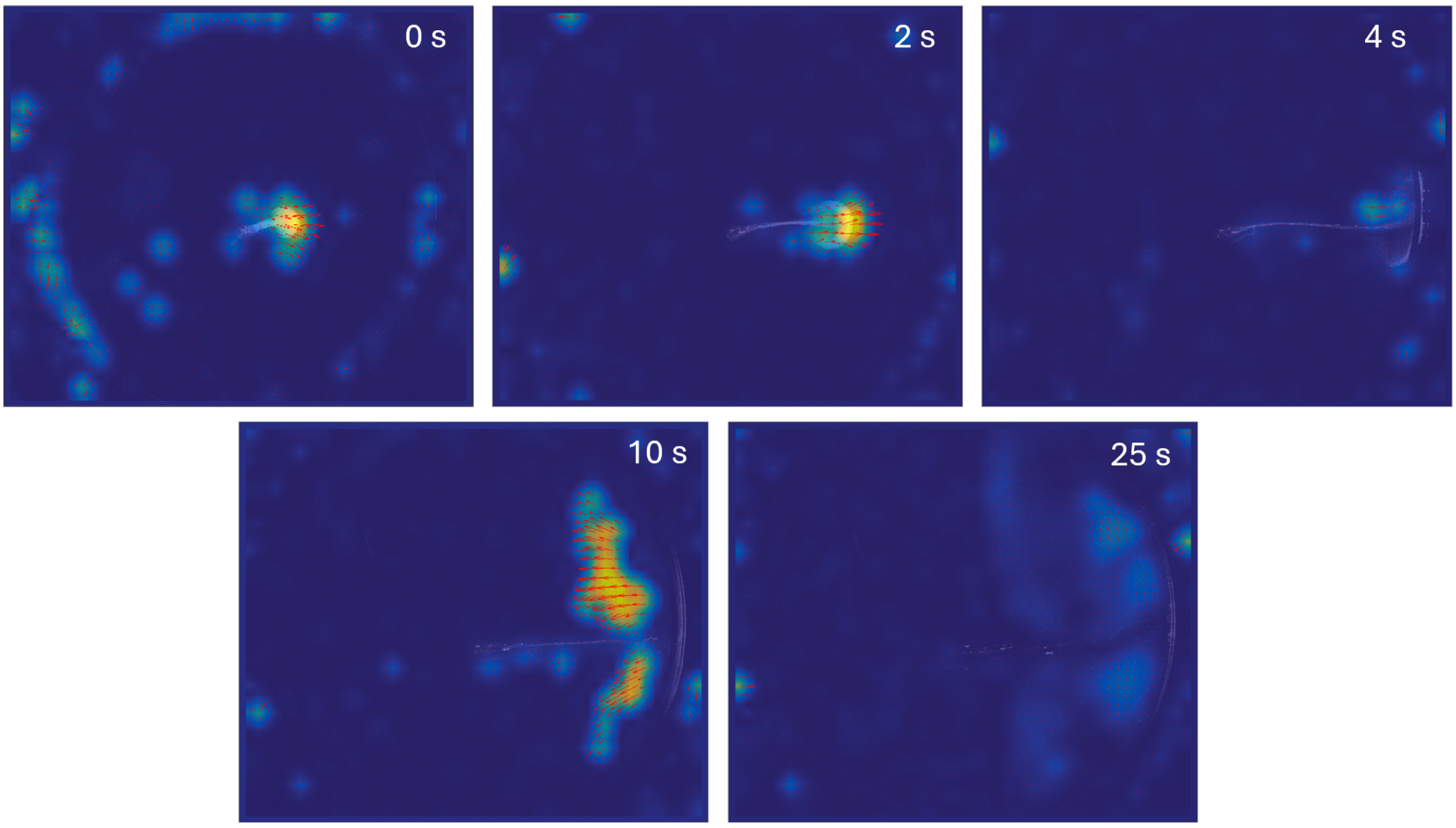

Figure S 7: Exploratory PIV analysis of active NM swarms under a magnetic field gradient. PIV analysis of the active NM swarm in urea under a magnetic gradient pointing toward the right side of the Petri dish, corresponding to the intensity images shown in Figure 5C. Red arrows indicate the local velocity field, and the color map indicates velocity magnitude. During the first seconds, the velocity field shows motion predominantly toward the right side, consistent with magnetic localization of the NM swarm, which reaches the Petri dish wall after approximately 4 s. After 10 s, the catalytic response induces redistribution of the NM cloud, with velocity components extending away from the target region and partially against the magnetic pulling direction, consistent with catalysis-triggered interfacial spreading. At 25 s, residual motion remains visible at the target site. This analysis is used as qualitative support for the magnetic-localization/catalytic-redistribution sequence, rather than as the primary quantitative comparison across conditions.

References

[1] Nian-Sheng Cheng. Formula for the viscosity of a glycerolwater mixture. *Industrial & Engineering Chemistry Research*, 47(9):3285–3288, 2008.

[2] A. C. Bakenecker, J. Schumacher, P. Blümler, K. Gräfe, M. Ahlborg, and T. M. Buzug. A concept for a magnetic particle imaging scanner with halbach arrays. *Physics in Medicine & Biology*, 65(19):195014, 2020.
