# Supplementary material for: Synergistic magneto-enzymatic propulsion enables multimodal nanomotor swarms
Source: Nat Commun. 2026 Aug 11;17:9660. doi: 10.1038/s41467-026-76053-x (PMC13558676; doi:10.1038/s41467-026-76053-x)
Supplement: Supplementary file 2 [file 41467_2026_76053_MOESM2_ESM.pdf]

File name: Video 1 - Motion behavior urea concentration
Description: corresponding to Fig.2 and S1: Motion behavior of the swarm at different urea concentrations

File name: Video 2 - Motion behavior particle concentration
Description: corresponding to Figure S2: Motion behavior of the swarm at different particle concentrations

File name: Video 3 - Motion behavior viscosity
Description: corresponding to Figure S3 and S4: Motion behavior of the swarm in different viscosities of the media

File name: Video 4 - Nanomotors vs passive particles
Description: corresponding to Figure 3: Comparison of active nanomotors with passive nanoparticles from top and side view

File name: Video 5 - Vortex Ring Theory
Description: corresponding to Figure 4: Vortex ring theory

File name: Video 6 - Collective dissipation in magnetic field
Description: corresponding to Figure 5: Magnetic actuation of magnetomotors in open Environment

File name: Video 7 - Magnetic actuation in straight channel from top view
Description: corresponding to Figure 6: Magnetic actuation of magnetomotors in straight channels observed from top view inside Halbach ring for 2mm and 4mm channel width

File name: Video 8 - Magnetic actuation in straight channel side view
Description: corresponding to Figure 6: Magnetic actuation of magnetomotors in straight channels observed from side view pulled by a single permanent magnet for 2mm channel width

File name: Video 9 - Magnetic actuation in L-shaped channel
Description: corresponding to Figure 6: Magnetic actuation of magnetomotors in L-shaped channels observed from top view inside Halbach ring for 2mm channel width
