# Supplementary material for: Synergistic magneto-enzymatic propulsion enables multimodal nanomotor swarms
Source: Nat Commun. 2026 Aug 11;17:9660. doi: 10.1038/s41467-026-76053-x (PMC13558676; doi:10.1038/s41467-026-76053-x)
Supplement: Supplementary file 3 [file 41467_2026_76053_MOESM4_ESM.pdf]

nature portfolio

Peer Review File

Synergistic Magneto-Enzymatic Propulsion Enables Multimodal Nanomotor Swarms

Corresponding Author: Professor Anna Bakenecker

This file contains all reviewer reports in order by version, followed by all author rebuttals in order by version.

Version 0:

Reviewer comments:

Reviewer #1

(Remarks to the Author)
The manuscript presents an innovative and visually compelling multimodal strategy that integrates magnetic guidance with enzymatic propulsion using magnetosome-based nanomotors. The ability to magnetically navigate the nanomotors to a target location and subsequently induce a Marangoni-driven expansion is conceptually novel. Overall, the work is promising, and with appropriate clarification and additional discussion addressing the points below, the manuscript will be significantly strengthened; therefore, I recommend it for acceptance.
1. The authors correctly point out that the Marangoni-driven interfacial expansion occurs at the air–water interface. It would be highly valuable for the readership if the authors could provide a more in-depth discussion regarding the relevance of this mechanism in realistic biomedical environments, which are typically fully liquid-filled or involve liquid–solid interfaces (e.g., blood vessels, urinary tract, or bladder). Specifically, the authors are encouraged to clarify whether analogous expansion or spreading behaviors could arise in confined liquid-phase environments, or to discuss how the proposed “multimodal” advantages might be realized in vivo if classical Marangoni effects are absent.
2. The observation of vortex-ring-like collective motion following nanomotor injection is intriguing. To further substantiate that this behavior originates from the intrinsic self-propulsion and collective dynamics of the nanomotors, rather than from transient hydrodynamic effects associated with the injection process, additional clarification would be beneficial.
3. Figure 1 could be further enriched to convey more information, especially by clearly illustrating the overall working principle of the system and highlighting the main conceptual innovations of this work.
4. With respect to quantitative analysis, the use of pixel-intensity-based “coverage area” as a metric for diffusion is understandable but may be sensitive to variations in illumination conditions, background subtraction, and swarm thickness. To improve the robustness of the analysis, it would be helpful if the authors could validate these results using complementary metrics, such as integrated fluorescence intensity or other density-independent quantification methods, thereby reinforcing the reliability of the reported diffusion behavior.

(Remarks on code availability)

Reviewer #2

(Remarks to the Author)
The manuscript reports on urea-powered nanomotors based on biogenic magnetosomes that undergo collective upward motion and horizontal expansion. Notably, the author uses naturally produced magnetosomes from magnetotactic bacteria and well characterizes the 3D motion via combined top/side views. The integration of fuel supply and external magnetic field control provides potential biological and environmental applications. Here are some comments for further consideration:
1. The urease–magnetosome nanomotors appear quite asymmetric in morphology, so net propulsion under urea fuel would be expected. However, the measured diffusion coefficients remain low and only weakly enhanced over Brownian motion. Could the author explain the suppressed motility?
2. The upward motion of NM is characterized via intensity projections. For urea = 50 mM group (purple line in Fig. 2C, D), the intensity distributions seem to be inconsistent. The data also appears to be noisy in Fig. 2E.
3. In Fig. S1a, NP in 500 mM urea solution also show spreading. How does it happen?
4. When referring to “particle swarms,” one usually expects some intrinsic interaction/communication between particles leading to synergic collective behavior. Can author add some discussion or analysis to strengthen the concept of swarming

instead of directed motion under external stimuli.
5. pH change is attributed to the critical process of the Marangoni-driven interfacial expansion. Could the author characterize the pH change caused by the catalytical reaction under experimental conditions? while directly measuring surface tension variations may be difficult.
6. Regarding NM swarming behaviors, has the author attempted to quantify the velocity fields, for example using PIV (particle image velocimetry) or related methods? Such analysis could help characterize collective motions.

(Remarks on code availability)

Reviewer #3

(Remarks to the Author)
In this work, the author developed an enzyme-functionalized magnetic nanomotor. The nanomotor swarm can be steered by magnetic field and performs catalytic expansion in urea solution. Several phenomena observed in the propulsion of the swam are recorded and analyzed. I believe the multimodal swarming phenomenon described in this working is interesting, the overall structure of this work is good and the figure presentation is attractive.However, I also have major concerns regarding this work.

1. The title mentions multimodal nanomotor swarms, but the term “multimodal” is not clearly defined. The author should clarify whether multimodal refers to locomotion modes or swarm configurations.

2. The most concerning question is the motivation is not clear. The author claims to investigate a new type of hybrid nanomotor swarm, but the advantage of this approach is not stated. The swarm behavior is not new, and the synergy between propulsion methods is not significant. If I understand correctly, I cannot find new mechanism or theory proposed. The authors shall largely enhance this part. Several swarming phenomena are recorded and hypotheses are proposed, but the core findings are hard to identify. The author needs to elaborate on the key insights.

3. Urease is functionalized via non-covalent bonding. The stability of this functionalization is not clearly stated or evaluated. The authors need to provide a proper evaluation method for it.

4. The author attributes catalytic expansion to deprotonation and surface activation. Does this process deactivate the nanomotor? A dynamics analysis is needed to show how the expansion evolves over time.

5. The vortex ring theory depicted in Fig. 4 involves dropping the swarm with a pipette. This seems irrelevant to the main claim of this paper. The author should either explain what insight this provides or reorganize this section.

6. The main advantage of this nanomotor swarm is not convincing. Fig. 6 suggests velocity is the advantage, but the speed enhancement is minorly observed. For instance, magnetic actuation can achieve higher speeds of microrobots.

(Remarks on code availability)

Version 1:

Reviewer comments:

Reviewer #1

(Remarks to the Author)
The authors have adequately addressed all of my concerns. I am satisfied with their responses and agree that the manuscript is suitable for publication.

(Remarks on code availability)

Reviewer #2

(Remarks to the Author)
The authors have provided a clear response to the previous comments, addressing most of my questions. I believe the article is now well-organized and will be of interest to the community.

(Remarks on code availability)

Reviewer #3

(Remarks to the Author)
The authors have addressed all my questions. I recommend for publication at the current form.

(Remarks on code availability)

Open Access This Peer Review File is licensed under a Creative Commons Attribution 4.0 International License, which permits use, sharing, adaptation, distribution and reproduction in any medium or format, as long as you give appropriate credit to the original author(s) and the source, provide a link to the Creative Commons license, and indicate if changes were made.
In cases where reviewers are anonymous, credit should be given to 'Anonymous Referee' and the source.
The images or other third party material in this Peer Review File are included in the article's Creative Commons license, unless indicated otherwise in a credit line to the material. If material is not included in the article's Creative Commons license and your intended use is not permitted by statutory regulation or exceeds the permitted use, you will need to obtain permission directly from the copyright holder.
To view a copy of this license, visit https://creativecommons.org/licenses/by/4.0/

Response to the Reviewers

Dear Reviewer,

We thank you for your careful and constructive reports. Your comments are reproduced below in full, followed by our point-by-point responses. For clarity, the modified text in the revised manuscript and Supporting Information is highlighted in red. We believe that the revisions have substantially improved the clarity, scope, and mechanistic framing of the manuscript, and we hope that the revised version will now be suitable for publication.

More in detail, we have:

1. defined "multimodal" more clearly as referring to distinct collective operating modes rather than multiple single-particle propulsion engines;
2. moderated broad application claims while preserving the broader environmental and biomedical motivation of the work;
3. strengthened the distinction between urea-driven buoyant accumulation and catalysis-dependent interfacial expansion;
4. added a pH characterization experiment to support the catalytic mechanism of the swarm;
5. clarified the quantitative analysis framework, emphasizing the robustness and limitations of the image-based metrics, the consistency across multiple complementary observables;
6. applied a PIV analysis as qualitative support for the magnetic-localization/catalytic-redistribution sequence;
7. repositioned the vortex-ring-like dynamics as a secondary injection-seeded hydrodynamic transport feature, rather than a central claim or novelty of the work; and
8. experimentally assessed the robustness of the non-covalent urease functionalization, including its functional retention over the experimental timescale through repeated washing, swarm operation, and post-swarm catalytic reassessment.

Sincerely,

Anna C. Bakenecker, Xavier Arqué and Samuel Sánchez
on behalf of all authors

Reviewer #1

Reviewer comment (general assessment)

The manuscript presents an innovative and visually compelling multimodal strategy that integrates magnetic guidance with enzymatic propulsion using magnetosome-based nanomotors. The ability to magnetically navigate the nanomotors to a target location and subsequently induce a Marangoni-driven expansion is conceptually novel. Overall, the work is promising, and with appropriate clarification and additional discussion addressing the points below, the manuscript will be significantly strengthened; therefore, I recommend it for acceptance.

Response

We sincerely thank the reviewer for this very positive and encouraging assessment of our manuscript. We particularly appreciate the recognition of the conceptual novelty of combining magnetic localization with catalysis-triggered interfacial expansion, as well as the reviewer's constructive suggestions. We have carefully addressed all the points raised below and believe that the revised manuscript is substantially strengthened as a result.

Comment 1

1. The authors correctly point out that the Marangoni-driven interfacial expansion occurs at the air–water interface. It would be highly valuable for the readership if the authors could provide a more in-depth discussion regarding the relevance of this mechanism in realistic biomedical environments, which are typically fully liquid-filled or involve liquid–solid interfaces (e.g., blood vessels, urinary tract, or bladder). Specifically, the authors are encouraged to clarify whether analogous expansion or spreading behaviors could arise in confined liquid-phase environments, or to discuss how the proposed "multimodal" advantages might be realized in vivo if classical Marangoni effects are absent.

Response

We thank the reviewer for this important comment and fully agree that the physical scope and application relevance of the interfacial mechanism should be discussed more carefully. In response, we have revised the Abstract, the final paragraph of the Introduction, the confinement discussion, and the Conclusions to clarify that the classical Marangoni-driven expansion reported here is strongest at open air–water interfaces and is therefore not meant to be presented as a generic mechanism for fully liquid-filled in vivo environments.

We now state more clearly that in fully confined liquid-phase environments, the same interfacial expansion is not expected to operate in the same way, which is consistent with our own confinement experiments showing that the expansion is suppressed while magnetic localization and local enrichment remain operative. At the same time, we note explicitly that urease nanomotors have already shown collective behavior in urea-rich settings such as the bladder, although through a mechanism distinct from the experimental setup with an air-water interface described here. We therefore cite the bladder application from the literature in the revised manuscript as an example of a relevant urea-rich biomedical setting, while clarifying that the present work addresses a different, interface-dependent collective mode.

Rather than claiming a specific near-term application, we now frame the present study as a fundamental step toward future environmental and biomedical implementations of multimodal nanomotor swarms. In this sense, the main contribution of the work is to establish the distinct collective mechanism itself, together with the physical conditions under which it emerges or is suppressed. We believe this more general framing preserves the broader motivation of the work while avoiding overstatement of immediate application scope.

Changes in manuscript: We revised the Abstract, pp. 1–2, lines 1–21; the Introduction discussion of biomedical relevance, p. 2, lines 49–58; the final paragraph of the Introduction, p. 3, lines 75–103; the mechanistic discussion distinguishing buoyant accumulation from catalysis-dependent interfacial expansion, p. 8, lines 231–248; the confinement discussion, p. 15, lines 432–438; and the Conclusions, p. 16, lines 439–461.

Comment 2

2. The observation of vortex-ring-like collective motion following nanomotor injection is intriguing. To further substantiate that this behavior originates from the intrinsic self-propulsion and collective dynamics of the nanomotors, rather than from transient hydrodynamic effects associated with the injection process, additional clarification would be beneficial.

Response

We agree that the origin and interpretation of the vortex-ring-like motion should be clarified more carefully. In the revised manuscript, we have revised Section 2.3 and the Figure 4 caption to distinguish more explicitly between: (i) the initial injection-seeded hydrodynamic structure, (ii) the urea-driven buoyant support that allows this structure to persist and evolve, and (iii) the catalysis-dependent interfacial expansion.

We now present these as successive components of the full mechanistic picture of the system. In this framework, the injection-seeded hydrodynamic redistribution and the urea-enabled buoyant support are not the main points of the work, but they are still important mechanistic insights because they contribute to explain the prerequisites under which the later magnetic localization and catalytic expansion can occur.

More specifically, we now clarify that the vortex-ring-like structure is interpreted as an early hydrodynamic transport mode that is supported by injection. Importantly, it is not observed in water, where the particles simply sediment, and it only develops in urea-containing media, where the buoyancy effect is sufficient to sustain the injected cloud and allow the ring-like dynamics to emerge and evolve. We therefore do not interpret this feature either as a trivial pipetting artefact or as a standalone signature of autonomous self-propulsion, but rather as a hydrodynamic mode seeded by injection and enabled by urea-induced buoyancy. In the active NM condition, catalysis only becomes relevant later, once the cloud reaches the air-water interface and undergoes the interfacial expansion described in the main mechanistic sections.

We have also clarified in the revised manuscript why this analysis can still be relevant from an application perspective: many realistic deployment scenarios for nanomotors involve bolus injection or catheter-based administration, in which an initially injected cloud may undergo similar early-stage hydrodynamic redistribution before subsequent magnetic localization or catalytic interfacial spreading. This point is now stated explicitly in the revised Section 2.3 and reflected in the updated Figure 4 caption.

At the same time, we made clear that the central contribution of the manuscript remains the catalytic swarm mechanism itself, while the vortex-ring discussion provides mechanistic context for how the particle cloud evolves immediately after injection.

Changes in manuscript: We revised the mechanistic discussion of the vortex-ring-like behavior in Section 2.3, pp. 10–11, lines 286–329, and the Figure 4 caption, p. 11.

Comment 3

3. Figure 1 could be further enriched to convey more information, especially by clearly illustrating the overall working principle of the system and highlighting the main conceptual innovations of this work.

Response

We thank the reviewer for this helpful suggestion and fully agree. In the revised manuscript, we have expanded Figure 1 to include an explanatory system-level schematic, effectively serving as a graphical abstract-like introductory panel, and revised its caption to provide the reader with a clearer overview of the system and of the intended collective operating principle. We also added explanatory text at the end of the Introduction to explicitly connect this schematic to the mechanistic questions that are tested later in the manuscript.

Because Figure 1 appears before the mechanistic results are presented, we were careful not to overstate findings that are only established later in the paper. Accordingly, the revised schematic is used primarily to introduce the system design and the proposed two-step collective concept, while more specific interpretations of urea-driven buoyant accumulation, and catalysis-triggered interfacial expansion is developed and justified later in the manuscript.

Changes in manuscript: We revised Figure 1 and its caption, p. 4, and added explanatory text connecting the schematic to the paper's mechanistic aims in the final paragraph of the Introduction, p. 3, lines 75–103.

Comment 4

4. With respect to quantitative analysis, the use of pixel-intensity-based "coverage area" as a metric for diffusion is understandable but may be sensitive to variations in illumination conditions, background subtraction, and swarm thickness. To improve the robustness of the analysis, it would be helpful if the authors could validate these results using complementary metrics, such as integrated fluorescence intensity or other density-independent quantification methods, thereby reinforcing the reliability of the reported diffusion behavior.

Response

In the revised manuscript, we have clarified the rationale, robustness, and limitations of the pixel-intensity-based "coverage area" analysis in the Methods. In particular, we now state that the videos are first subjected to background correction, which makes the analysis robust against moderate illumination inhomogeneities, and that the subsequent binarization above a fixed threshold makes the coverage-area readout relatively insensitive to illumination differences within the non-saturated and non-underexposed regime used in our experiments.

We also emphasize in Sections 2.2 and 2.3 that the main conclusions are not based on this metric alone, but are supported jointly by coverage area, FWHM, center-of-

mass evolution, and the corresponding top/side-view image series. Although these observables are all extracted from the same image datasets and can each be influenced to some extent by the evolving projected thickness of the swarm, they probe different aspects of the redistribution dynamics. Their interpretation is therefore strengthened by the combined top/side-view analysis, which helps distinguish lateral spreading from vertical accumulation and changes in cloud thickness. Rather than relying on a single additional metric, we now make clearer in the manuscript that the conclusions derive from the consistency across these complementary observables, interpreted together with the image sequences.

We agree that a direct comparison with integrated fluorescence intensity would be valuable. However, the present experiments were performed with non-labelled magnetosome nanomotors and were recorded using light-intensity-based imaging; therefore, the same experiments cannot be quantitatively reanalyzed in fluorescence without a separate dual-channel or fluorescence-labelled acquisition. Our goal was to establish a robust analysis workflow for the available light-intensity videos, using background correction, threshold-based area detection, and complementary observables to compare the different experimental conditions consistently.

We have therefore revised the text to make clear that the reported trends rely on the consistency across multiple observables and on a robust preprocessing workflow, rather than on a single image-derived metric in isolation.

Changes in manuscript: We clarified the image-analysis workflow and its limitations in the Methods, pp. 18–19, lines 562–592. We also clarified in the Results that the conclusions are supported by multiple observables, particularly Section 2.3, p. 10, lines 277–285, and Section 2.4, p. 12, lines 364–384.

Reviewer #2

Reviewer comment (general assessment)

The manuscript reports on urea-powered nanomotors based on biogenic magnetosomes that undergo collective upward motion and horizontal expansion. Notably, the author uses naturally produced magnetosomes from magnetotactic bacteria and well characterizes the 3D motion via combined top/side views. The integration of fuel supply and external magnetic field control provides potential biological and environmental applications. Here are some comments for further consideration:

Response

We thank the reviewer for the careful reading of the manuscript and for recognizing the originality of using biogenic magnetosomes, the value of the combined top/side-view 3D characterization, and the interest of integrating enzymatic activity with magnetic control. We have carefully revised the manuscript in response to the comments below.

Comment 1

1. The urease–magnetosome nanomotors appear quite asymmetric in morphology, so net propulsion under urea fuel would be expected. However, the measured diffusion coefficients remain low and only weakly enhanced over Brownian motion. Could the author explain the suppressed motility?

Response

We agree that the structural asymmetry of the urease–magnetosome nanomotors could in principle support single-particle ballistic propulsion. However, at the nanoscale, the effective diffusion of an active particle reflects not only catalytic propulsion, but also its passive Brownian motion and, importantly, its orientational persistence. In very small particles, the passive diffusivity is relatively large, but rotational randomization is also much faster, so directional persistence is rapidly lost. As a result, even when catalytic asymmetry is present, a ~50 nm particle can still display only a modest enhancement over Brownian diffusion. In this sense, the relatively limited increase that we observe is not inconsistent with the size of the system.

A useful way to interpret this behavior is to compare both the absolute diffusion coefficients and the relative enhancement upon fuel addition with previous urease-powered nanomotor systems. Considering the range from the lowest tested non-zero urea concentration to the highest concentration yielding the maximum

diffusion coefficient, representative literature examples show increases approximately from 1.1 to 1.3 $\mu m^2 s^{-1}$ (+18%, *Llopis-Lorente et al., ACS Nano 2019*), from 0.8 to 1.1 $\mu m^2 s^{-1}$ (+38%, *Hortelão et al., ACS Nano 2019*), and from 0.9 to 1.2 $\mu m^2 s^{-1}$ (+33%, *Hortelão et al., Adv. Funct. Mater. 2018*). In our urease–magnetosome nanomotors, the measured increase is from approximately 1.5 to 2.2 $\mu m^2 s^{-1}$, corresponding to about +47%. Thus, although our system displays a higher absolute diffusivity, which is also reasonable given its much smaller size compared with the previously reported 250–400 nm urease nanomotors, the observed relative enhancement remains within a plausible range for urease-powered nanosystems.

Importantly, this distinction is itself central to the message of the paper: rather than focusing on strong enhancement of single-particle diffusivity, we investigate how weak individual nanoscale motility can give rise to a much more powerful emerging collective behavior, namely a catalysis-triggered expansion at the swarm level, and how this can be combined with magnetic localization into a two-step multimodal strategy. In this sense, the work is centered on the transition from limited individual motion to functionally useful collective dynamics, enabled by the interaction of many nanomotors with the surrounding medium, the interface, and an applied magnetic field.

We have therefore clarified in the revised manuscript, immediately after the DLS discussion in Section 2.1, that the key focus of the work is not single-particle propulsion performance, but the emergence of a collective magneto-catalytic behavior that becomes relevant at the swarm level.

Changes in manuscript: We added this clarification after the DLS analysis in Section 2.1, p. 5, lines 136–146. This revision also includes additional references to previous urease-powered nanomotor reports showing comparable diffusion enhancement, p. 5, lines 143–144, specifically references [42, 68, 69].

Comment 2

2. The upward motion of NM is characterized via intensity projections. For urea = 50 mM group (purple line in Fig. 2C, D), the intensity distributions seem to be inconsistent. The data also appears to be noisy in Fig. 2E.

Response

We carefully re-examined the relevant image series and analysis, particularly Video 1 and the corresponding data in Figure 2C–E. The snapshots shown in Figure C are consistent with the projection profiles in panel D, showing that only the NM in 100 mM and 500 mM move upwards to the surface. Only in these conditions we observe a spreading of NM at the surface, which can be seen in panel E in the distribution

profile. The 50 mM condition we interpret as an intermediate buoyancy regime, in which the data suggest that the nanomotor population splits into two subpopulations: one fraction behaves predominantly as a sedimenting population, while another fraction has access to the uplifting effect of urea. As a result, the cloud does not reorganize as efficiently into either a strongly sedimented or a strongly uplifted state, which reduces the net spreading in both directions. At the same time, because a significant fraction of nanomotors remains in the bulk for longer times, the corresponding intensity projections can exhibit a higher and narrower peak, consistent with a thinner and more concentrated intensity distribution.

We also acknowledge that video-specific artefacts, such as occasional bubbles in some recordings, can contribute to local fluctuations in the extracted intensity plots. However, after re-examining the corresponding videos and quantitative profiles, these artefacts appear limited to specific frames or regions and do not account for the overall concentration-dependent behavior observed across the urea series. We therefore interpret them as minor experimental fluctuations rather than the main origin of behavior in the 50 mM urea condition. We have therefore revised the text in Section 2.2 accordingly.

Changes in manuscript: We revised the discussion of the 50 mM urea condition in Section 2.2, p. 6, lines 175–183, where we now describe this condition as an intermediate buoyancy regime rather than simply as a noisy trace.

Comment 3

3. In Fig. S1a, NP in 500 mM urea solution also show spreading. How does it happen?

Response

Although this distinction was already introduced in the main text in Section 2.2 and further discussed mechanistically in Section 2.3, we should state this more explicitly for Figure S1. In the revised manuscript, we now explain more clearly that inactive NP in urea can still undergo upward accumulation and broad redistribution at the interface due to extreme urea-driven buoyant/solutal transport, even in the absence of catalysis. At very high urea concentration (500 mM), this buoyancy effect is sufficiently strong that the NP cloud reaches the interface already broadly distributed, without the need for catalytic activity. This has been also observed by Chen et al. (*Nat. Commun*. 2024).

Importantly, this behavior does not correspond to an evolving catalytic expansion front. Rather, the NP cloud appears at the interface in a broadly redistributed state,

and does not undergo the later abrupt interfacial spreading event observed for active nanomotors in urea. This distinction is evident in Video 1, where the passive NP at 500 mM urea (Figure S1A,C) show immediate broad accumulation at the interface, whereas the active NM in urea show the onset of expansive behavior only in the catalytically active conditions shown in Figure 2 and, in more detail, in Video 4 (Figure 3). We have therefore revised the text to distinguish more clearly between strong urea-driven interfacial broadening and true catalysis-triggered interfacial expansion.

Changes in manuscript: We revised the discussion of inactive NP behavior at high urea concentration in Section 2.2, pp. 5–6, lines 163–169, and further clarified the distinction between buoyant accumulation and catalytic expansion in Section 2.3, p. 8, lines 231–248. We have also added a clarification in the caption of Figure S1.

Comment 4

4. When referring to “particle swarms,” one usually expects some intrinsic interaction/communication between particles leading to synergic collective behavior. Can author add some discussion or analysis to strengthen the concept of swarming instead of directed motion under external stimuli.

Response

We agree that the term “swarming” can carry different connotations in different communities. In the present context, we use it in the broader active-matter sense to denote a global collective behavior without centralized control, where the dynamics of the ensemble emerge through coupling with the surrounding physicochemical environment rather than necessarily through direct communication between individual particles.

In the present system, the relevant interactions are mediated by the medium. In particular, we state that the collective behavior described here emerges at the ensemble level from particle accumulation, buoyancy-driven redistribution, interfacial Marangoni flow, and magnetic localization. More specifically, urea concentration gradients generate local density differences that promote buoyant uplift of the injected cloud, whereas ureolysis modifies the interfacial composition and pH, creating surface-tension gradients that drive outward Marangoni flow along the air–water interface. Thus, the collective behavior arises through particle–environment coupling, rather than through direct particle–particle signaling.

In the revised manuscript, we now clarify this point more explicitly in the Introduction. We have also refined the wording throughout the manuscript to

distinguish more carefully between direct particle–particle communication and collective dynamics emerging from particle–environment coupling.

Changes in manuscript: We clarified our use of “swarming” in the Introduction, p. 2, lines 35–43, and further specified particle cloud–environment coupling in the final Introduction paragraph, p. 3, lines 83–89.

Comment 5

5. pH change is attributed to the critical process of the Marangoni-driven interfacial expansion. Could the author characterize the pH change caused by the catalytical reaction under experimental conditions? while directly measuring surface tension variations may be difficult.

Response

Following the referee’s suggestion, we have now added a pH characterization experiment under the relevant experimental conditions. In this assay, the same media compositions used in the swarm experiments were prepared, and the pH evolution was monitored after adding a small volume of urease-functionalized magnetosome nanomotors to a 100 mM urea solution, exactly as in the previously reported experiments. As controls, we monitored the pH evolution of 100 mM urea alone and of magnetosomes in the absence of urea. The measurements were performed using a GLP21 pH meter equipped with an Orion-Ross pH-Micro electrode.

The results show that the NM + urea condition exhibits a clear pH increase (up to pH 8.5 within 15-20 min), whereas the 100 mM urea solution alone and the magnetosome control remain nearly unchanged at a pH level of 6.6-6.8. This provides direct experimental support that ureolysis by the urease-functionalized magnetosomes produces the alkalinization invoked in our mechanism. We agree that this bulk pH measurement does not directly resolve the transient local pH gradients at the air–water interface, and we state this explicitly in the manuscript. Importantly, because the measurement reports the bulk pH of the solution, the slower rise observed over 15–20 min likely underestimates the faster and higher local alkalinization expected in the immediate vicinity of the nanomotors and at the air–water interface during the early stages of swarm expansion. Thus, while the assay does not provide a direct local interfacial pH map, it confirms that the condition producing the catalytic interfacial expansion is also the condition that generates a measurable ureolysis-driven pH increase.

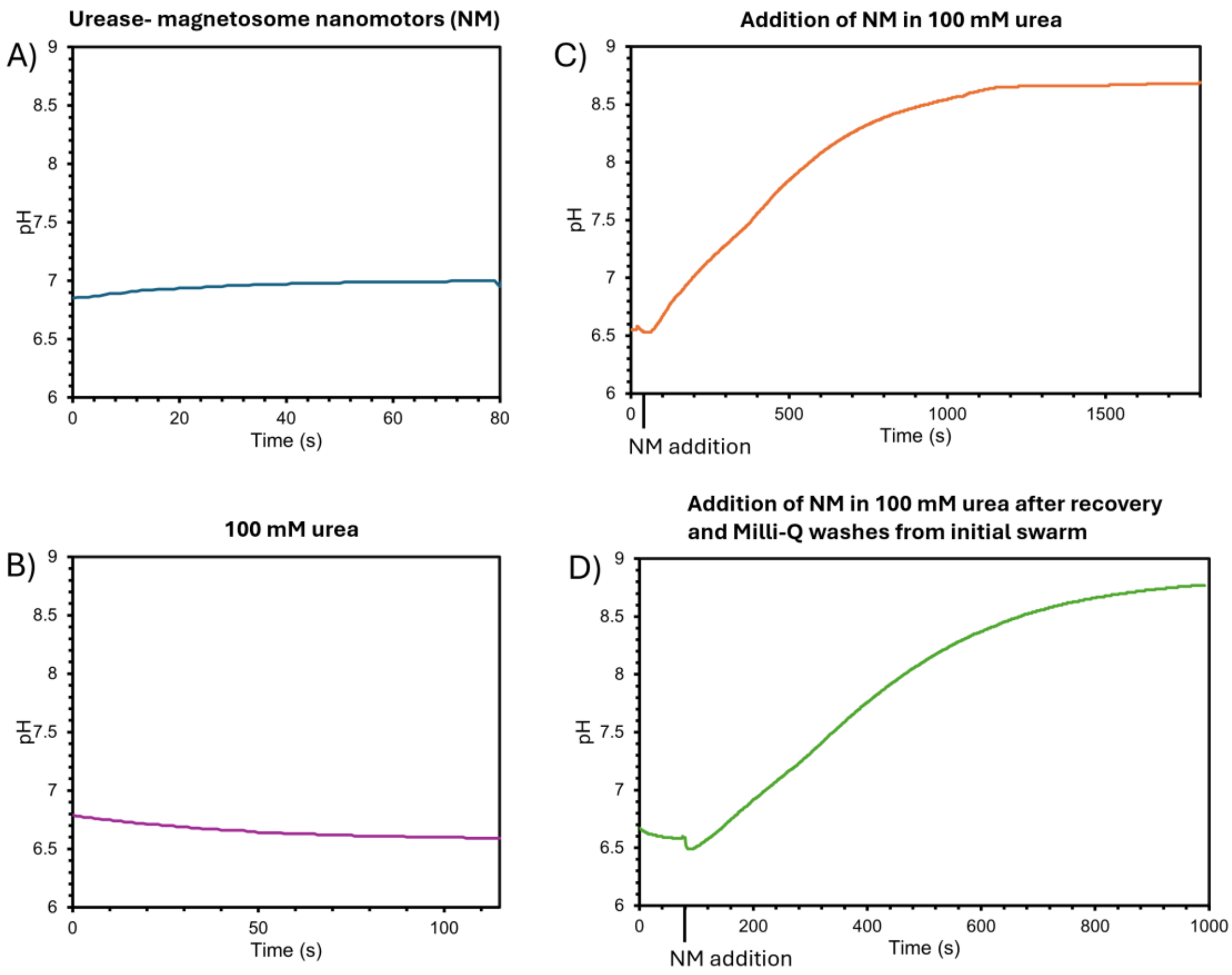

Figure S5: pH characterization of urease-functionalized magnetosome nanomotors and retained catalytic activity after recovery. Time evolution of pH for control and catalytic conditions. A) Magnetosomes in water over time. B) 100 mM urea solution over time. C) Urease-functionalized magnetosome nanomotors added to 100 mM urea solution producing a clear pH increase, supporting ureolysis-driven alkalinization under swarm-relevant conditions. D) After the swarm experiment, the nanomotors were recovered by centrifugation, washed x3 times with Milli-Q, and re-exposed to fresh 100 mM urea solution. The recovered nanomotors still induce a clear pH increase, indicating retained catalytic activity after swarm operation and recovery. (Figure incorporated into Supporting Information)

We also note that this interpretation is consistent with previous urease-powered micro/nanomotor studies in which catalytic activity gives rise to local pH changes or pH-mediated functional effects. For example, Patiño et al. (*Nano Lett.*, 2019) used pH-responsive DNA nanoswitches to sense and respond to the local alkalinization generated by ureolytic activity, while Arqué et al. (*Research*, 2020) showed that the ionic species produced during urea hydrolysis play a central role in propulsion and environmental modulation. In a related biomedical setting, Choi et al. (*Bioactive Materials*, 2022) showed that urease micromotors can locally increase pH and thereby induce gel–sol transition of the mucus layer to facilitate penetration.

Most importantly in the present context, Chen et al. (*Nat. Commun.*, 2024) demonstrated that urease swarms display collective behavior associated with the products of ureolysis in a buoyancy-driven mechanism. Although the interface-dependent Marangoni mechanism reported here is distinct, these studies provide a clear precedent for pH modulation as a functionally relevant mechanism in urease-powered systems, including at the collective/swarm level.

We have therefore revised the mechanistic section in Section 2.3, added the new pH characterization and retained-activity data as Supplementary Figure S5, and included the corresponding experimental procedure in the Methods.

Changes in manuscript: We revised the mechanistic discussion of pH-mediated catalytic activity in Section 2.3, p. 8, lines 249–260. This includes added literature support for pH-mediated functional effects in urease-powered micro/nanomotor systems, including references [71] and [72], p. 8, lines 251–255, as well as the urease-swarm precedent in reference [73], p. 8, lines 256–260. We also added the new pH characterization and retained-activity experiment in Section 2.3, p. 8, lines 261–269, included the corresponding data in Supplementary Figure S5, and added the experimental procedure in the Methods, p. 19, lines 593–608.

Comment 6

6. Regarding NM swarming behaviors, has the author attempted to quantify the velocity fields, for example using PIV (particle image velocimetry) or related methods? Such analysis could help characterize collective motions.

Response

We thank the reviewer for this valuable suggestion. We agree that velocity-field analysis would be informative. At the same time, for dense and deformable swarm clouds such as those studied here, there is no single standardized analysis method that can be considered a universal gold standard, particularly when the recorded signal corresponds to a 2D projection of a 3D evolving particle distribution. Our intention in this work is therefore to provide a robust framework to compare conditions consistently, rather than to rely on a velocity-field method whose interpretation is ambiguous for the present datasets.

More specifically, the image signal arises from a dense and evolving particle distribution rather than a passively advected tracer field. The swarm occupies a finite thickness along the optical axis, so the image corresponds to a 2D projection of a 3D cloud, where overlap along z, out-of-plane motion, and changes in local cloud thickness can alter the signal independently of in-plane transport. In addition, the particle cloud undergoes strong deformation during uplift, spreading, and ring

formation. For these reasons, conventional 2D tracer-based PIV was not used as the primary quantitative tool for comparing conditions, while image intensity can still be informative of redistribution and concentration changes.

Following the reviewer's suggestion, we nevertheless performed an exploratory PIV analysis of the image sequence shown in Figure 5C. This qualitative analysis supports the main interpretation of the magnetic experiment: during the first seconds, the velocity field points predominantly toward the right side of the Petri dish, consistent with magnetic localization of the NM swarm toward the applied field gradient. After the onset of the catalytic response, the velocity field changes and shows redistribution away from the localized region, including motion components opposite to the magnetic pulling direction, consistent with catalysis-triggered interfacial spreading at the target site. Thus, the PIV analysis qualitatively reinforces the conclusion that the system first undergoes magnetic localization and then catalytic redistribution.

We therefore use PIV only as a supportive qualitative analysis, while keeping our main comparison framework based on observables that can be robustly extracted and quantitatively traced across all conditions, namely covered area, FWHM, center-of-mass displacement, vertical intensity projections, and the corresponding top/side-view image sequences. We have clarified this methodological choice in the revised Methods and provide the exploratory PIV analysis in Supplementary Figure S7.

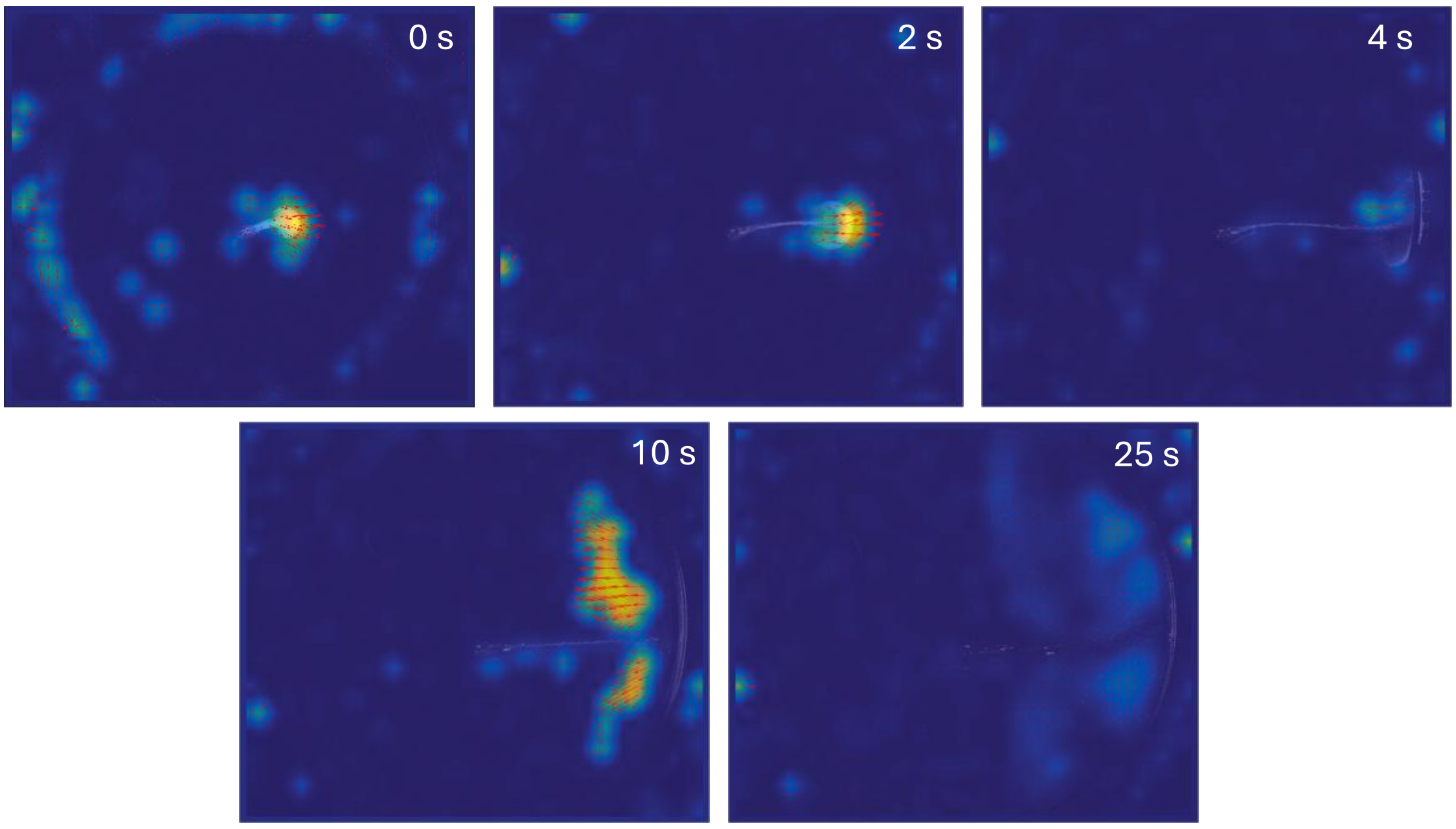

Figure S7: Exploratory PIV analysis of active NM swarms under a magnetic field gradient. PIV analysis of the active NM swarm in urea under a magnetic gradient pointing toward the right side of the Petri dish, corresponding to the intensity images shown in Figure 5C. Red arrows indicate the local velocity field, and the color map indicates velocity magnitude. During the first seconds, the velocity field shows

motion predominantly toward the right side, consistent with magnetic localization of the NM swarm, which reaches the Petri dish wall after approximately 4 s. After 10 s, the catalytic response induces redistribution of the NM cloud, with velocity components extending away from the target region and partially against the magnetic pulling direction, consistent with catalysis-triggered interfacial spreading. At 25 s, residual motion remains visible at the target site. This analysis is used as qualitative support for the magnetic-localization/catalytic-redistribution sequence, rather than as the primary quantitative comparison across conditions. (Figure incorporated into Supporting Information)

Changes in manuscript: We clarified the quantitative image-analysis framework in the Methods, pp. 18–19, lines 562–592. We also added an exploratory PIV analysis as qualitative support in Section 2.4, p. 12, lines 375–384, included the corresponding data in Supplementary Figure S7, and added the PIV procedure in the Methods, pp. 19–20, lines 609–623.

Reviewer #3

Reviewer comment (general assessment)

In this work, the author developed an enzyme-functionalized magnetic nanomotor. The nanomotor swarm can be steered by magnetic field and performs catalytic expansion in urea solution. Several phenomena observed in the propulsion of the swam are recorded and analyzed. I believe the multimodal swarming phenomenon described in this working is interesting, the overall structure of this work is good and the figure presentation is attractive. However, I also have major concerns regarding this work.

Response

We thank the reviewer for the careful evaluation of our manuscript and for recognizing the interest of the multimodal collective phenomenon, the overall structure of the manuscript, and the figure presentation. We also appreciate the critical comments, which helped us identify where the central message and scope of the manuscript required substantial clarification. In response, we have revised the manuscript extensively to better define the main conceptual contribution and to avoid overstating claims that are not central to the work.

Comment 1

1. The title mentions multimodal nanomotor swarms, but the term "multimodal" is not clearly defined. The author should clarify whether multimodal refers to locomotion modes or swarm configurations.

Response

We understand the term "multimodal" may need further clarification. In the revised manuscript, we now define "multimodal" more explicitly as referring to two sequential and complementary collective operating modes of the swarm: (i) magnetic localization / concentration of the nanomotor ensemble; and (ii) catalysis-triggered interfacial expansion of that localized ensemble.

This clarification now appears in the revised Abstract and Introduction.

Changes in manuscript: We defined "multimodal" in the Abstract, pp. 1–2, lines 7–20, and in the final paragraph of the Introduction, p. 3, lines 75–80.

Comment 2

2. The most concerning question is the motivation is not clear. The author claims to investigate a new type of hybrid nanomotor swarm, but the advantage of this approach is not stated. The swarm behavior is not new, and the synergy between propulsion methods is not significant. If I understand correctly, I cannot find new mechanism or theory proposed. The authors shall largely enhance this part. Several swarming phenomena are recorded and hypotheses are proposed, but the core findings are hard to identify. The author needs to elaborate on the key insights.

Response

We thank the reviewer for this important and constructive comment. However, we respectfully note that our interpretation of the novelty is more specific than a generic claim of "new swarming." In response, we have substantially rewritten the Abstract, Introduction, Results framing, and Conclusions to define the novelty more precisely and to avoid overstating the scope of the work.

More specifically, we now clarify that the novelty we claim is not that "swarming" itself is new. Rather, the manuscript advances two main contributions. First, it identifies a distinct catalytic swarm mechanism, namely:

1. urea-driven buoyant accumulation of the particle cloud toward the air–water interface, followed by
2. catalysis-triggered interfacial Marangoni expansion of that accumulated cloud.

To the best of our knowledge, previous urease nanomotor swarm studies have reported buoyancy-driven collective motion, but not this specific two-stage catalytic collective mechanism. This distinction was already present in the originally submitted manuscript, particularly in the mechanistic discussion of Section 2.3, where we contrasted the present mechanism with prior systems showing urea-induced buoyant rise without interfacial expansion, and with the recent free-urease interfacial Marangoni study, which is mechanistically related but does not involve a nanomotor swarm platform. In the revised manuscript, we have now made this distinction much more explicit earlier in the text, especially in the final paragraph of the Introduction and in the Conclusions.

Second, we now make clearer that the present system also represents, to the best of our knowledge, the first combination of a catalytically driven swarm mechanism with magnetic guidance at the collective level. We consider this important not simply because two modes are combined, but because their interplay is non-trivial:

(i) Catalytically propelled active motion typically lacks intrinsic directionality, yet directional control is crucial for targeted applications and for minimizing the required agent loading. Magnetic guidance is particularly well suited to address this limitation.

(ii) At the same time, the study is significant from a fundamental perspective, because our results reveal a counterintuitive finding: the Marangoni-driven interfacial expansion can overcome the opposing magnetic attraction. This interplay enables the swarm to be guided directionally to a specific location while preserving its ability to expand over the target area upon arrival. In this regard, the added value of the hybrid strategy is not merely the integration of multiple motion mechanisms, but the non-trivial combination of global steerability with local ensemble-level dispersion.

The key insights are now stated as:
(i) the identification of a distinct catalytic collective mode,
(ii) the demonstration of a non-trivial magnetic–catalytic swarm combination, and
(iii) the definition of the threshold conditions under which these behaviors emerge or are suppressed, including particle loading, urea concentration, viscosity, confinement, and magnetic configuration.

Changes in manuscript: We revised the Abstract, pp. 1–2, lines 1–21; the final paragraph of the Introduction, p. 3, lines 75–103; the single-particle versus collective-motion framing in Section 2.1, p. 5, lines 136–146; the viscosity-dependent quenching of the Marangoni expansion in Section 2.2, p. 6, lines 198–215; the mechanistic distinction between buoyant accumulation and catalysis-dependent interfacial expansion in Section 2.3, p. 8, lines 231–248; the magnetic-guidance section, pp. 12–13, lines 330–384; the confinement discussion, p. 15, lines 385–438; and the Conclusions, p. 16, lines 439–461.

Comment 3

3. Urease is functionalized via non-covalent bonding. The stability of this functionalization is not clearly stated or evaluated. The authors need to provide a proper evaluation method for it.

Response

We first wish to clarify that the aim of the present study is to establish functional stability on the timescale of the swarm experiments, rather than long-term storage stability.

Importantly, as stated in the manuscript, the nanomotors are subjected to multiple washing steps after functionalization in PBS and again in Milli-Q water before use,

while still retaining catalytic activity during the swarm experiments. In this sense, the experimental workflow itself already indicates that the non-covalently adsorbed urease remains functionally associated with the magnetosomes throughout repeated washing and use. To address this point more directly, we have now added a post-swarm recovery assay. After the initial pH characterization experiment, the nanomotors were recovered by centrifugation, washed x3 times by removing the supernatant and resuspending the particles in Milli-Q water, and then re-exposed to a fresh 100 mM urea solution. The recovered nanomotors again produced a clear pH increase upon renewed addition to urea, showing that urease remained functionally active and associated with the magnetosomes after prior operation, recovery, and washing.

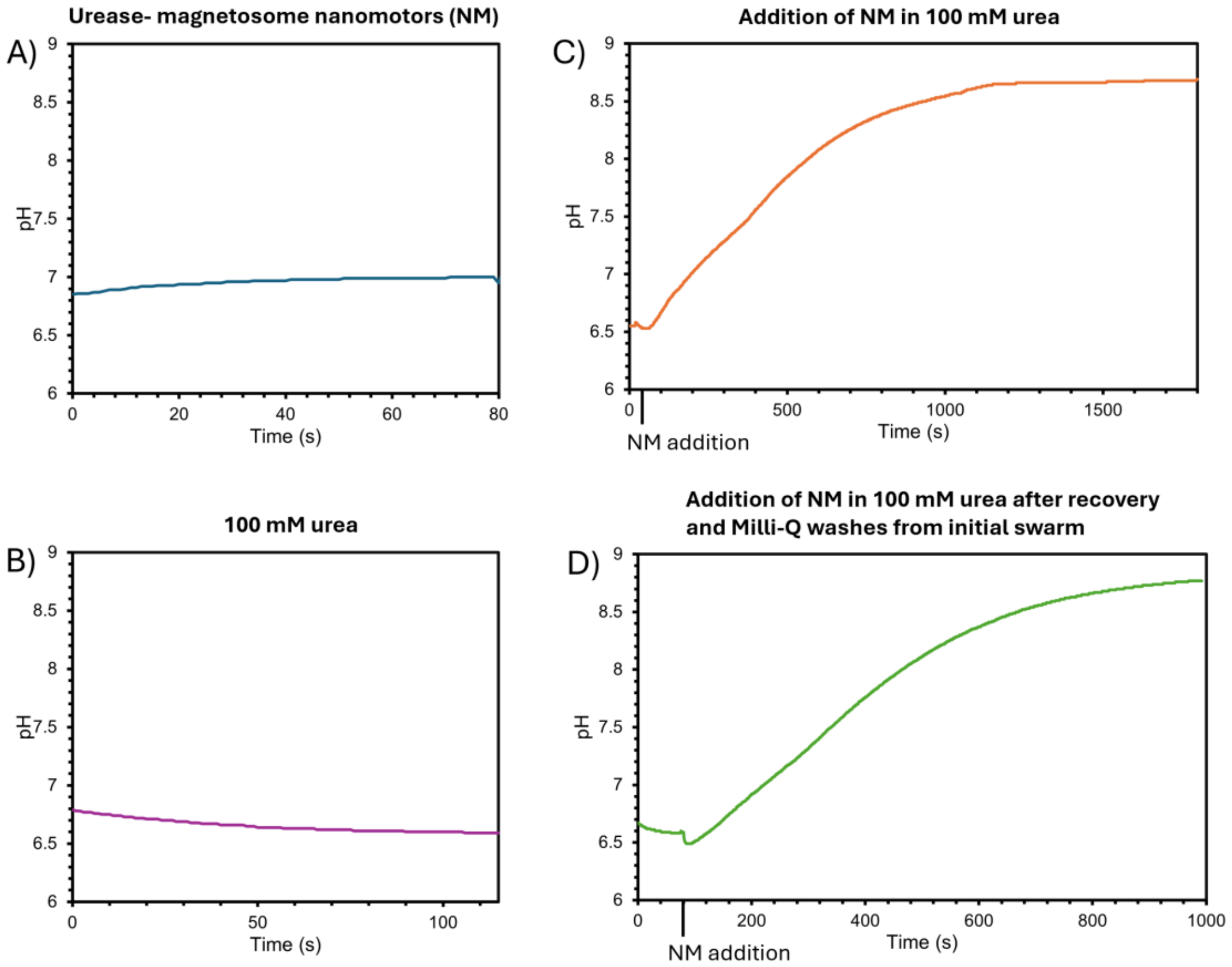

Figure S5: pH characterization of urease-functionalized magnetosome nanomotors and retained catalytic activity after recovery. Time evolution of pH for control and catalytic conditions. A) Magnetosomes in water over time. B) 100 mM urea solution over time. C) Urease-functionalized magnetosome nanomotors added to 100 mM urea solution producing a clear pH increase, supporting ureolysis-driven alkalinization under swarm-relevant conditions. D) After the swarm experiment, the nanomotors were recovered by centrifugation, washed x3 times with Milli-Q, and re-exposed to fresh 100 mM urea solution. The recovered nanomotors still induce a clear pH increase, indicating retained catalytic activity

after swarm operation and recovery. (Figure incorporated into Supporting Information)

This result is now reported in Supplementary Figure S5. In the revised manuscript, we refer to this assay both in the Methods, at the end of the functionalization paragraph, where we clarify that non-covalent urease attachment remains functionally stable over the experimental timescale and repeated washing steps, and in Section 2.3, where we state that the retained catalytic response after recovery supports continued functional association of urease with the magnetosomes after swarm operation.

We now use this result to clarify that, in the present work, enzymatic activity is assessed through the catalytic pH response, which provides a direct functional readout of urease retention over the experimental timescale relevant to the swarm measurements. It demonstrates that sufficient urease remains associated to preserve catalytic activity after the swarm reaction, repeated washing and recovery.

Changes in manuscript: We clarified the urease-functionalization procedure and functional-retention assessment in the Methods, p. 17, lines 505–514. We also added the post-swarm recovery pH assay and retained-activity interpretation in Section 2.3, p. 8, lines 261–269, included the corresponding data in Supplementary Figure S5, and added the experimental procedure in the Methods, p. 19, lines 593–608.

Comment 4

4. The author attributes catalytic expansion to deprotonation and surface activation. Does this process deactivate the nanomotor? A dynamics analysis is needed to show how the expansion evolves over time.

Response

We thank the reviewer for raising these two mechanistic points. We have clarified both: the possible effect of the expansion process on nanomotor activity and the time-resolved nature of the expansion in the revised manuscript.

First, regarding possible nanomotor deactivation, we do not expect the interfacial expansion process itself to produce an immediate loss of nanomotor function on the experimental timescale considered here. To address this point directly, we refer to the same post-swarm recovery experiment described in response to Comment 3: after the swarm experiment, the nanomotors were recovered, washed again, and re-exposed to urea, and their catalytic response was reassessed through a pH evolution assay. The recovered samples still showed a clear pH increase, indicating that the interfacial expansion does not cause an immediate loss of enzymatic

activity under our experimental conditions. This result is now reported in Supplementary Figure S5 and referenced in the Methods and Section 2.3.

Second, regarding the dynamics of the expansion, the temporal evolution of the expansion is analyzed throughout the manuscript using the time-dependent covered-area and FWHM plots, as well as the corresponding top/side-view image sequences. These datasets quantify the latency before expansion, the rate of spreading, and the final extent of the expanded cloud under the different experimental conditions. In addition, the time evolution can be directly followed in the corresponding videos and in the associated intensity projections, particularly in Videos 5 and 6, where the redistribution of the cloud is resolved dynamically rather than only through endpoint measurements.

In the revised manuscript, we now highlight this more explicitly in Sections 2.2–2.4 and state that the expansion evolves as a time-resolved collective process, rather than as a static endpoint observation. Combined with the new recovery pH assay, these results indicate that the catalytic expansion is associated with a transient interfacial collective process and not with immediate nanomotor deactivation.

Changes in manuscript: We clarified the temporal evolution of the expansion in Section 2.2, p. 6, lines 170–185; Section 2.3, pp. 8–10, lines 270–329; and Section 2.4, pp. 12–13, lines 356–384. We also added literature support for pH-mediated urease activity and its functional consequences in Section 2.3, p. 8, lines 249–260, including references [71] and [72]. Finally, we added the post-swarm recovery pH assay used to address possible deactivation in Section 2.3, p. 8, lines 261–269, Supplementary Figure S5, and Methods, p. 19, lines 593–608.

Comment 5

5. The vortex ring theory depicted in Fig. 4 involves dropping the swarm with a pipette. This seems irrelevant to the main claim of this paper. The author should either explain what insight this provides or reorganize this section.

Response

We agree with the reviewer that the vortex-ring-like dynamics needed to be more clearly positioned with respect to the main claim of the manuscript. In the revised manuscript, we have rewritten Section 2.3 and the Figure 4 caption to make clear that the vortex-ring-like behavior is not the central claim or novelty of the manuscript, but rather an early hydrodynamic transport mode that helps contextualize the redistribution of the injected particle cloud.

We now clarify that the vortex-ring-like structure is initiated by injection, but it is not observed in water, where the particles simply sediment. It only develops in urea-

containing media, where the buoyancy effect is sufficient to sustain the injected cloud and allow the ring-like dynamics to emerge and evolve.

We believe it remains useful to discuss this behavior because it contributes to explain the full evolution of the system after injection, before the later stages of magnetic localization and catalysis-driven interfacial expansion become dominant. We also note that many realistic deployment scenarios for nanomotors involve bolus injection or catheter-based administration, in which similar early-stage hydrodynamic redistribution may be relevant before the subsequent collective modes take over.

Changes in manuscript: We reorganized and reframed the vortex-ring-like behavior in Section 2.3, pp. 10–11, lines 286–329, and revised the Figure 4 caption, p. 11.

Comment 6

6. The main advantage of this nanomotor swarm is not convincing. Fig. 6 suggests velocity is the advantage, but the speed enhancement is minorly observed. For instance, magnetic actuation can achieve higher speeds of microrobots.

Response

We thank the reviewer and agree that this distinction needed to be clarified more explicitly. In the revised manuscript, we now separate the work into two operational scenarios, and we make clearer that the manuscript is primarily a mechanistic study of how enzymatic and magnetic capabilities combine at the swarm level, rather than a claim of superior speed performance.

In the open-interface Petri-dish experiments (diameter 3 cm), which constitute the core setting of the manuscript, the main advantage is not velocity, but the ability to first magnetically localize the swarm to a chosen region and then catalytically expand it across a target area at the air–water interface. In this setting, the key function of the system is therefore directionality combined with local interfacial coverage, rather than enhanced translational speed. We now emphasize this as the main advantage of the nanomotor swarm in the revised Section 2.4 and in the corresponding discussion of the Halbach-ring experiments.

By contrast, Figure 6 corresponds specifically to confined-channel transport, where the free-interface expansion is suppressed and the transport behavior under confinement is compared. In that context, the speed difference is not intended as the core conclusion of the paper, and we agree that it should not be interpreted as evidence of a general superiority over purely magnetic microrobots. Rather, these experiments were included to examine how different degrees of confinement hinder

or modify the full swarm behavior. The confined results therefore serve mainly to show that the more open the environment, the more successfully the combined magnetic–catalytic swarm behavior can emerge, whereas confinement progressively suppresses the expansive interfacial mode. This clarification now appears in the revised confinement discussion.

Accordingly, in the revised manuscript we no longer present speed as the general advantage of the platform. Instead, we emphasize that the central contribution of the work is the study of a combined magnetic–enzymatic swarm behavior that can provide both on-demand directionality and targeted expansion, together with an analysis of the conditions under which this behavior is enabled or hindered.

Changes in manuscript: We revised the magnetic-guidance section to emphasize directionality and target-area coverage rather than speed, pp. 12–13, lines 330–384. We also reframed the confinement experiments as a boundary-condition study rather than a speed-performance claim, including the interpretation of faster magnetic steering in wider channels, p. 15, lines 385–407, and the final confinement framing, p. 15, lines 432–438. This interpretation was consolidated in the Conclusions, p. 16, lines 439–461.
